# Process evaluation of Project Daire: a food environment intervention that impacted food knowledge, wellbeing and dietary habits of primary school children

**DOI:** 10.1186/s12889-025-21628-4

**Published:** 2025-02-06

**Authors:** Naomi Anderson, Sarah F. Brennan, Fiona Lavelle, Sarah E. Moore, Dilara Olgacher, Amy Junkin, Moira Dean, Michelle C. McKinley, Patrick McCole, Ruth F. Hunter, Laura Dunne, Niamh E. O’Connell, Chris T. Elliott, Danielle McCarthy, Jayne V. Woodside

**Affiliations:** 1 Institute for Global Food Security, Queen’s University Belfast, Belfast, BT9 5AG UK; 2 Centre for Public Health, Queen’s University Belfast, Belfast, BT12 6BA UK; 3 Department of Nutritional Sciences, School of Life Course & Population Sciences, King’s College London, London, UK; 4 School of Social Sciences, Education and Social Work, Queen’s University Belfast, Belfast, UK; 5 Queen’s Management School, Queen’s University Belfast, Belfast, BT9 5EE UK; 6 Maynooth University, Maynooth, Co. Kildare Ireland

**Keywords:** Process evaluation, School, Children, Diet, Food, Education, Childhood wellbeing, Child behaviour, Food environment, Whole-school approach

## Abstract

**Background:**

Project DAIRE was a randomised-controlled, factorial design trial which aimed to improve children’s health-related quality of life, wellbeing, food knowledge and dietary habits via two multi-component interventions: Nourish and Engage. Nourish was an intervention aiming to alter the school food environment, provide food-based experiences and expose pupils to locally produced foods. Engage was an age-appropriate cross-curricular food education intervention incorporating food, agriculture, nutrition science and related careers. The purpose of this study was to conduct a process evaluation to evaluate DAIRE implementation, mechanisms of impact (MOI) and context to elucidate trial results, and inform scalable implementation of the DAIRE approach for successful future rollout.

**Methods:**

The Medical Research Council’s (MRC) framework for process evaluation was followed. Formal (questionnaires designed for process evaluation) and informal (researcher records and communications) methods were used to collect quantitative and qualitative data during the DAIRE trial in relation to process evaluation. Quantitative data were analysed using descriptive statistics and qualitative data via thematic analysis to identify key themes.

**Results:**

Fifteen schools and 983 pupils (*n* = 495 6–7 year olds/Year 3 and *n* = 488 10–11 year olds/Year 7) were recruited for the 6-month DAIRE intervention; a 100% retention rate was observed at the school level and the interventions had a high level of pupil and teacher acceptability. Nourish schools delivered a higher mean dose of intervention elements (61.4%) than Engage (50%) schools but, overall, mixed implementation of both interventions occurred. DAIRE produced change through four key MOI: social learning, experimental learning, interactive engaging content and real-life connections. Lack of time was the main contextual barrier to implementation and lack of financial cost to schools indicated as a potential facilitator.

**Conclusions:**

This process evaluation helped to identify important findings related to implementation, MOI and context. The most effective elements of the interventions which should be maintained include provision of interactive and engaging intervention elements at no financial cost to the school. Findings also identified suggestions for improvement including provision of increased teacher training, support and planning time, content reduction to facilitate easy integration, and implementation across the full academic year. A sustainable funding and resourcing mechanism is required for successful future roll-out across the UK and beyond.

**Trial registrations:**

The original trial referenced in this process evaluation is registered as follows: National Institute of Health (NIH) U.S. National Library of Medicine Clinical Trials.gov (ID: NCT04277312; retrospectively registered 11th February 2020).

**Supplementary Information:**

The online version contains supplementary material available at 10.1186/s12889-025-21628-4.

## Background

The link between childhood diet and disease prevention in adulthood is well established and research suggests that eating patterns track from childhood to adulthood [1–3]. Consequently, establishing healthy dietary patterns in childhood is critical to influencing future disease risk and adult health status [1–3]. Schools have been recognized as ideal environments for public health interventions [4] due to the proportion of time children spend in school and the significant number of meals consumed in this setting. As such, calls for action to policy makers to improve the dietary quality of food provided in the school setting has intensified [5, 7] and a whole-school approach advocated [5].

In Northern Ireland, the primary school curriculum incorporates 11 areas of learning, and there is scope to cover aspects of food and health within the ‘Personal Development and Mutual Understanding’ and ‘The World Around Us’ Areas of Learning [6], but this is at the discretion of teachers and schools and it is difficult to get a sense of to what extent food and health is covered across the primary school curriculum. As such, DAIRE undertook a multi-stakeholder approach to improve primary school children’s knowledge of and interest in food with an aim of improving health-related quality of life and wellbeing via two 6-month multi-component interventions. DAIRE was a school-based randomised-controlled, factorial design four-arm trial conducted in Northern Irish (NI) primary school settings [8]. DAIRE tested elements of the whole school approach through two interventions – Nourish and Engage which aimed to improve primary school children’s knowledge of and interest in food. Nourish was a whole-school food environment intervention aiming to alter the current school food environment and focused on food provision, resources to improve school food presentation and the school food environment, play-based food-related experiences and exposure to locally produced foods. The Nourish intervention aimed to influence childhood awareness of food groups, encourage tasting and identification of new foods, improve dietary intake and awareness of food preparation and cooking techniques and was informed by pre-intervention observations conducted at schools in the region which captured information on current practices related to the school food environment. Engage was an age-appropriate cross-curricular food education intervention, which involved educational and nutrition-related science and career food topics such as the food chain, product development, growing food, animal welfare, sustainability, food labels, portion size, diet and health. Engage consisted of interactive lessons and engaging food experiences in the community and incorporated aspects of the current Northern Ireland Curriculum including literacy, mathematics and physical activity. Activities were supported by lesson plans, videos, books, worksheets, games, visits and practical experiments. Engage was developed by the research team in conjunction with stakeholders, including primary school teachers and local food producers, who helped advise on type of content, integration of content within the current curriculum and tailoring of content to ensure age-appropriate [8]. Project DAIRE worked in partnership with primary schools and a range of stakeholders, including teachers, principals, school caterers and local food producers, to develop and deliver the interventions. The study demonstrated that improvements in childhood emotional and behavioural wellbeing, dietary intake, knowledge about food, cooking skills and willingness to try new foods were associated with the ‘Nourish’ whole-school food environment intervention [8].

Schools-based interventions are complex and process evaluation (PE) is an essential part of designing and testing complex interventions, as it can enable researchers to assess whether interventions were delivered as intended and elucidate causal mechanisms and contextual factors that may be associated with any variation in outcomes. As such, results from PEs can inform policy makers on how best to implement health interventions in different population groups or contexts [9, 10]. Knowledge gained from PEs of school-based interventions have offered valuable information for future implementation of similar trials and the potential effectiveness of whole-school and multilevel approaches. For example, a recent PE of a primary-school Food Education and Sustainability Training (FEAST) program in Australia reported that if intervention duration increased and if supported by school policies, such interventions have the potential to improve children’s dietary intake, cooking skills and food waste behaviours [11]. Further to this, a PE of a gamified school-based intervention that aimed to promote healthy dietary intake and sustainable behaviours in high school students in Italy highlighted that increased dosage and additional activities may be warranted even though the trial supported the use of gamified approach [12].

Due to the complex nature of DAIRE and to fully understand its outcomes, we have conducted a process evaluation using both quantitative and qualitative data collected throughout the DAIRE study and following the MRC framework to evaluate DAIRE implementation, mechanisms of impact (MOI) and context [8–10].

The objectives were:

1. To evaluate implementation –through evaluating fidelity, acceptability, dose, reach, recruitment, retention and contamination of both the Nourish and Engage interventions.
2. To evaluate the MOI of both the Nourish and Engage interventions.
3. To evaluate context of the Nourish and Engage interventions and determine which contextual factors hindered and supported effective implementation of DAIRE and influence intervention outcomes.
4. To provide scientific rationale for modifications to the Nourish and Engage interventions which will facilitate successful and effective future DAIRE

## Methodology

### DAIRE study design

DAIRE was a randomised-controlled, factorial design four-arm trial evaluating two interventions in the primary school setting in an area of socio-economic disadvantage in the north-west of Northern Ireland. The DAIRE study protocol and main outcomes are published elsewhere [8]. In summary, schools were randomised to one of four 6-month intervention arms: i) ‘Nourish’, ii) ‘Engage’, iii) ‘Nourish’ and ‘Engage’ and iv) Control (Delayed) and data were collected both pre and post-intervention. DAIRE worked in partnership with primary schools and a range of stakeholders, including teachers, principals, school caterers and local food producers, to develop interventions for pupils in year groups aged 6–7 and 10–11 years to improve knowledge of, and interest in, food. A total of 15 schools were randomized to one of the four intervention arms: i) ‘Nourish’ (n = 4), ii) ‘Engage’ (n = 3), iii) ‘Nourish and Engage’ (n = 4) and iv) ‘Control’ (Delayed) (n = 4). The specific components of the interventions are outlined in Additional File 1. Ethical approval for DAIRE was obtained from the School of Social Sciences, Education and Social Work Ethics Committee, Queen’s University Belfast (Reference number 038_1819).

Participating schools were randomised to ‘Nourish’, ‘Engage’, ‘Nourish and Engage’ and Control (Delayed) arms after baseline data collection. The allocation sequence was produced in STATA using block sizes of n = 4. Schools were stratified by religious affiliation, because this is how schools are typically organised within the Northern Ireland school system, to ensure a balanced approach as described in main DAIRE trial paper [8].

Mainstream (non-special) schools were eligible to participate if they met the following criteria: schools willing to be randomly assigned to an intervention, those willing to engage with and implement the intervention with their pupils and those willing to facilitate data collection. Schools had to be located within the North-West region of Northern Ireland. A list of all mainstream primary schools in the region was obtained from the local council and schools were contacted and informed about the study. If schools expressed interest in taking part they were sent a Memorandum of Understanding (MoU) to consider and sign. Information sheets and consent forms were then sent to parents and a system of opt-out parental consent was in place.

### DAIRE process evaluation design, sample population and data collection

The DAIRE process evaluation design, key functions and interrelationships, adapted from Moore et al. [10] incorporated consideration of the implementation of both the Nourish and Engage interventions, the quantity and quality of what was delivered, mechanisms of impact in relation to participant responses to, interactions with and level of engagement with DAIRE interventions to produce outcomes, and context in relation to external factors which influenced DAIRE intervention outcomes, to obtain process evaluation outcomes.

#### Sample population

In total 15 schools and 983 pupils (*n* = 495 6–7 year-old Key Stage 1 (KS1) pupils (Year 3) and n = 488 10–11 year-old Key Stage 2 (KS2) pupils (Year 7)) were recruited for the DAIRE intervention. The total number of pupils (*n* = 494 males and n = 489 females) allocated to the DAIRE interventions were as follows i) Nourish (*n* = 189), ii) Engage (*n* = 244), iii) Nourish and Engage (*n* = 308) and iv) Control (Delayed) (*n* = 242). Additionally, three Irish language schools expressed interest in participating but, as it was not feasible in terms of time or funding to translate all intervention resources in the event that these schools were allocated to one of the Engage intervention arms, these schools were not randomized but received the Nourish intervention instead. School characteristics are presented in the DAIRE trial paper [8]. In summary, at baseline there were *n* = 550 male and *n* = 573 female pupils, *n* = 597 pupils aged 6–7 years, *n* = 526 pupils aged 10–11 years, n = 5 schools were in rural locations and *n* = 10 schools were in urban locations. Data were collected from *n* = 903 (*n* = 445 aged 6–7 years and *n* = 458 aged 10–11 years).

Outcome measures were translated to the Irish language and then back translated for the 3 Irish language schools to facilitate ecological validity as far as possible and enable children to complete in the appropriate language. As such, data collected from these schools were presented separately in the main paper [8].

#### Process evaluation data collection

Quantitative and qualitative process evaluation data were collected throughout DAIRE in both a formal and informal manner, as shown in Table 1. Table 2 outlines each DAIRE process evaluation element, how each element was investigated and data used to investigate each element.

**Table 1:** DAIRE process evaluation data collected

		**Data Type**	
		**Quantitative**	**Qualitative**
**Formal**	NEQ (completed by teachers)	✓	✓
	ETEQ (completed by teachers)	✓	✓
	NOT (completed by study researchers)	✓	✓
	EOT (completed by study researchers)	✓	✓
**Informal (study records)**	Nourish implementation summary document (study researcher records)	✓	X
	Nourish field notes records (study researcher records)	✓	X
	Nourish planned Tasting Days document (study researcher records)	✓	X
	Emails regarding healthy snack delivery (communication received from industry partners)	✓	✓
	Mobile Petting Farm visit records (communication received from company and study researcher records)	✓	X
	Engage schools records (study researcher records)	✓	X
	Completed lessons records (teacher and study researcher records)	✓	X
	DAIRE feedback and quotes document (study researcher records of verbal feedback received from teachers during data collection)	X	✓
	Overview of Engage delivery on DAIRE researcher document (teacher and study researcher records)	X	✓
	DAIRE guest speakers document (study researcher records)	X	✓
	Records on class perceptions of the completed PE lesson (study researcher records)	✓	✓
	Email communication on school recruitment (communication received from school management)	✓	✓
	Class lists and opt-outs information (communication received from school management)	✓	X
	Records of numbers of baseline/end-point pupils (study researcher records)	✓	X
	School food environment dataset (study researcher records)	✓	✓

*Abbreviations*: *NEQ* Nourish Evaluation Questionnaire, *ETEQ* Engage Teacher Evaluation Questionnaire, *NOT* Nourish Observation Tool, *EOT* Engage Observation Tool

**Table 2:** The DAIRE process evaluation framework and the data used to investigate each element

DAIRE process evaluation element		Data used to investigate		
		**Engage**	**Nourish**	**How each element was investigated**
**Implementation**	***Recruitment***	Email communication on school recruitment Class lists and opt-out information	Email communication on school recruitment Class lists and opt-out information	Expressed as the total number of pupils in each intervention arm and DAIRE as a whole
	***Retention***	Records of numbers of baseline/end-point pupils Class lists and opt-out information Complete responses data for HRQOL and SDQ scores	Records of numbers of baseline/end-point pupils Class lists and opt-out information Complete responses data for HRQOL and SDQ scores	Expressed as the mean % of opt-out pupils, as well as the % completion rate of complete (both baseline and endpoint) data responses for the KIDSCREEN-10 [13] questionnaire (and Strength and Difficulties (SDQ) [14] questionnaire
	***Reach*** *‘the extent to which a target audience comes into contact with the intervention’*	Email communication on school recruitment NOT – Q7, Q8, Q9 Class lists and opt-out information	Email communication on school recruitment EOT – Q6, Q7, Q8 Class lists and opt-out information	Using recruitment and retention data to determine the extent to which primary school children aged 6–7 and 10–11 years in the North West region of Northern Ireland came into contact with DAIRE
	***Fidelity**** And ****Dose*** *‘the consistency of what was implemented with the planned intervention’ and ‘the quantity of intervention delivered’*	NOT – Q1, Q2, Q4, Q5, Q6, Q8, Q10, Q11, Q14, Q15 NEQ – Q1, Q2, Q3, Q4, Q18 Nourish implementation summary document Nourish school field notes records Nourish planned Tasting Days document Emails regarding healthy snack delivery Complete responses data for HRQOL and SDQ scores	ETEQ – Q1, Q2, Q3, Q6, Q8, Q10 EOT –Q5, Q6, Q7, Q8, Q9. Q10, Q15, Q16 Mobile Petting Farm visits records Engage schools records Completed lessons records Complete responses data for HRQOL and SDQ scores	Nourish fidelity was determined by the % of Nourish elements delivered and made available to schools, and the % of Nourish elements delivered (dose) in each individual school Engage fidelity was determined as the % of Engage elements made available to schools, the mean % of topic 1, topic 2 and topic 3 delivered (dose) in Engage schools, and the mean % of guest speakers, visits and activities implemented in schools (dose)
	***Acceptability***	NOT – Q2, Q4, Q5, Q6, Q10, Q11, Q14, Q15 NEQ – Q5, Q6, Q7, Q9, Q10, Q14, Q15, Q17 DAIRE feedback and quotes document	ETEQ – Q4, Q5, Q7, Q9, Q11, Q15, Q16, Q18 Overview of Engage delivery on DAIRE document DAIRE feedback and quotes document DAIRE guest speakers document Process Evaluation class copy of perceptions dataset EOT –Q5, Q6, Q7, Q8, Q9. Q10, Q15, Q16	Participant responses to Likert-scale questions in the NEQ and ETEQ were coded on a 5-point scale ranging from 1 ‘Strongly Agree’ to 5 ‘Strongly Disagree’. Mean score, standard deviation, frequencies and %’s were calculated and expressed, with a lower score indicating higher acceptability Thematic analysis of informal data sources outlined in Table 1
	***Contamination*** *‘The likelihood of control group participants being exposed to the active intervention’*	Brennan et al. [7] main DAIRE paper: intervention study design, recruitment, randomisation and allocation	Brennan et al. [7] main DAIRE paper: intervention study design, recruitment, randomisation and allocation	Examining the likelihood of DAIRE control group participants being exposed to the Nourish and Engage interventions through examining the DAIRE intervention study design, recruitment, randomisation and allocation
***Mechanisms of impact*** *‘How did delivered intervention produce change (participant responses to and* *interactions with* *interventions, mediators, unexpected pathways and* *consequences)’*		NEQ – Q3, Q5, Q6, Q7, Q9, Q10, Q14 NOT – Q6, Q10, Q14 Junkin [16] Msci paper	ETEQ – Q4, Q5, Q7, Q9, Q11, Q15 EOT – Q5,Q9,Q15, Q16	Frequencies and %’s were calculated with regards relevant NEQ and ETEQ questions on pupil reaction to the interventions and which elements were most useful and appropriate (see Table 2) Thematic analysis of informal data sources outlined in Table 1
***Context*** *‘Factors external to the intervention which may influence implementation, or whether MOI act as intended’*		NEQ – Q3, Q11, Q12, Q13, Q16 School food environment dataset Nourish implementation summary document Complete responses data for HRQOL and SDQ scores Follow up questionnaire	ETEQ – Q3, Q6, Q10, Q12, Q13, Q14 Engage schools records School food environment dataset Completed lessons records Complete responses data for HRQOL and SDQ scores Follow up questionnaire	Frequencies and %’s were calculated with regards both the schools and teachers at classroom level who reported barriers, the presence of similar health initiatives and if substitute teachers were in place during intervention delivery Thematic analysis of informal data sources outlined in Table 1

#### Formal data collection

Process-Evaluation specific questionnaires were developed by the research team based on study protocol and informed by the MRC framework [9] and formed the majority of the structured formal data collection for the DAIRE PE.

Specifically, two teacher evaluation questionnaires were developed:

1. The Nourish Evaluation Questionnaire (NEQ) (Additional File 2).
2. The Engage Teacher Evaluation Questionnaire (ETEQ) (Additional File 3).

The questionnaires comprised of 18 questions and were designed to be administered to intervention schools only, with the ETEQ administered to primary school teachers and the NEQ administered to the most relevant school personnel within each school i.e., teaching staff, principal/vice principal, catering/canteen staff. Both the NEQ and ETEQ were administered at the end of the intervention period.

In addition, two observational tools were also developed, which were informed by the MRC framework [9]:

1. Nourish Observation tool (NOT) (Additional File 4).
2. Engage Observation tool (EOT) (Additional File 5).

The 15-item NOT and 16-item EOT were intended for use by DAIRE researchers to observe intervention lessons and activities in the field following school permission.

The questionnaires and observation tools were based on the three MRC framework elements and were designed to collect quantitative and qualitative information on: intervention delivery, appropriateness and usefulness of intervention elements, any adaptations that were made by teachers to intervention elements, whether substitute teachers were in place during intervention delivery, level of pupil engagement with the interventions, any barriers experienced during delivery, general opinions on the interventions and the presence of similar health initiatives in schools during the intervention period [9].

#### Informal data collection

Informal data, i.e. data collected naturally as part of the study (data not collected by the developed structured questionnaires), were also collected by DAIRE researchers at schools randomised to the intervention groups throughout the intervention period (see Table 1). Informal data included content and dates of relevant emails and other communications sent by school personnel, guest speakers and food industry partners regarding the Nourish and Engage interventions, and conversations and comments made during the data collection period.

### Follow-up interviews

The DAIRE team planned to conduct follow-up interviews with school staff about their views and experiences of DAIRE but were unable to do so due to COVID-19-related school closures. Instead, one year after completion of the study, follow-up questionnaires (Additional File 6) were emailed to the schools which were designed to be completed by school representatives (e.g. principals, teachers and/or caterers) and collected quantitative and qualitative data on the impact of DAIRE on individual school food policies, healthy snack provision, use of DAIRE components after the intervention period, and suggestions for future implementation of DAIRE.

#### Data analyses

Quantitative data were entered into SPSS and cleaned prior to analysis. Qualitative data were compiled, analysed and coded according to the specific element(s) of the DAIRE process evaluation framework they were relevant to. Table 2 presents the process evaluation framework, how each DAIRE process evaluation element was investigated and data used to investigate each element and statistical analyses used.

Quantitative questionnaire data (NEQ and ETEQ) were analysed using SPSS statistical analysis software V 27.0. Descriptive statistics (frequencies, means, ± standard deviation and percent values) were calculated to determine the implementation – specifically fidelity, dose, reach, recruitment, retention and acceptability, MOI and context aspects of the framework (Table 2). Thematic analysis of qualitative data was conducted following the Braun and Clarke [15] six step framework for the implementation – specifically fidelity, dose and acceptability, MOI, and context aspects of the framework (*see* Table 2*for data used*). Qualitative data included formal written data collected via NEQ (completed by teachers), ETEQ (completed by teachers), NOT (completed by study researchers), EOT (completed by study researchers) and written informal data collected via: emails regarding healthy snack delivery (communication received from industry partners), research records of written DAIRE feedback and quotes documented from teachers, written records related to Engage delivery from researchers and teachers, researcher records on DAIRE guest speakers, researcher records on class perceptions of completed PE lessons, email communication on school recruitment (communication received from school management) and researcher records on school food environment dataset (Table 2). Written data were reviewed by a study researcher line by line several times to become familiar with the overall scope of the qualitative data collected. Once data were reviewed, the researcher proceeded to perform the initial stage of inductive coding based on the frequency of key words, phrases, opinions and concepts contained within the data. The study researcher shared the first stage of coding with the wider research team to discuss and reach a consensus with regards to appropriateness of codes and whether there were sufficient supporting data for each theme. Themes were further critically developed and discussed within the research teams and any discrepancies regarding the codes and themes were resolved through team discussions to reach a consensus. The main over-arching themes were confirmed once all queries and nuances that arose during the process were agreed upon.

## Results

For the main DAIRE trial, thirty schools were assessed for study eligibility. Six schools did not meet inclusion criteria, six schools did not respond further and three Irish language schools were directly allocated to the ‘Nourish’ intervention and not randomised, as described in main trial publication. In total, 15 primary schools were randomized [8].

For PE, 51.6% (*n* = 16) of the NEQ and 42.3% (*n* = 11) of the ETEQ were returned by teachers. Only a small number of schools gave permission for observations to be conducted so 9.7% (*n* = 3) of the NOT and 9.1% (*n* = 1) of the EOT were completed by the research team.

### Implementation – recruitment

The DAIRE intervention recruitment process, which was completed over a 6-week period (Jan-Feb 2019) and resulted in 15 randomised schools, is available in Additional File 7.

### Retention

A 100% retention rate was observed at both school and pupil level, with no schools (*n* = 15) dropping out, or pupils withdrawing consent once the DAIRE intervention began. Only five schools provided information regarding individual numbers of pupils who opted-out (data on *n* = 128/983 pupils); indicative of 13% of pupils based on this subset. Retention was also determined in terms of the number of paired baseline and end-point responses for primary outcomes within the 15 randomised DAIRE schools (*n* = 983 pupils).

The rate of completion for the pupil- focused Strength and Difficulties questionnaire, completed by teachers, was much lower (54.2%; *n* = 533) than the pupil-completed questionnaire for the primary outcome, KIDSCREEN-10 questionnaire, with 79.7% (*n* = 783) completing both at baseline and end-point (13, 14).

### Reach

It was not feasible to assess the exact % of primary school children aged 6–7 and 10–11 years in the NW region of NI that DAIRE reached, as there are no publicly available data on the number of pupils by age group in the region. However, in total, DAIRE reached 983 primary school children (n = 495 aged 6–7 and n = 488 aged 10–11 years) and, considering a total of 142 mainstream primary schools in the NW region of NI were contacted, with an approach aiming to include schools in both urban and rural locations, both faith and non-faith based schools and those where English was not used as first language (Irish language schools), DAIRE recruited 10.6% (n = 15 randomised) of possible mainstream primary schools within this region.

### Fidelity and dose

#### Nourish intervention

In total, 100% (*n* = 8) of Nourish schools received intervention elements and resources to enable complete intervention delivery. An additional option to hold a school food event sponsored by food industry partners was offered to only *n* = 3 Nourish randomised schools due to practicalities and *n* = 2 availed of this opportunity. Table 3 highlights that, on average, Nourish schools (*n* = 8) implemented 61.4% of the Nourish intervention, (range of 28.6% school Q to 85.7% school G).

**Table 3:** Summary of Nourish intervention elements implemented

School Code	Nourish elements								Nourish elements delivered at individual school level *n (*%)
	*Healthy snack provision*	*Enhancement of dining area*	*Enhanced food presentation*	*Cookery equipment and recipes*	*Sensory education*	*Optimise school food policies*	*Tasting day*	School food event*	
**E**	✓	✓	✓	✓	X	X	✓	N/A	5(71.4)
**F**	✓	✓	✓	✓	X	X	✓	N/A	5(71.4)
**G**	✓	✓	X	✓	✓	✓	✓	N/A	6(85.7)
**J**	✓	✓	X	✓	X	X	✓	✓	5(62.5)
**L**	✓	X	✓	X	X	X	✓	✓	4(50)
**P**	✓	✓	✓	X	X	X	✓	X	4(50)
**Q**	✓	✓	X	X	X	X	X	N/A	2(28.6)
**R**	✓	X	✓	X	X	X	✓	N/A	3(42.9)
**Nourish schools who delivered element (n = 8)**	8(100)	6(75)	7(87.5)	4(50)	1(12.5)	1(12.5)	7(87.5)	2(66.7)	
**Mean Dose of Nourish elements delivered in schools (%):** 61.4									
**Mean (SD) number of Nourish elements delivered in schools:** 4.3 (1.28)									

^*^Only 2 schools offered this option. Note: N/A for school food event as schools not offered option to partake

The most delivered Nourish element was healthy snack provision, which 100% (*n* = 8) of Nourish schools availed of. A total of 75% (*n* = 6) and 87.5% (*n* = 7) of schools utilised equipment to enhance the school environment and presentation of food respectively, and 50% (*n* = 4) of schools utilised cookery equipment and recipes. A high percentage of 87.5% (*n* = 7) of schools attended Tasting Days. One school utilised the sensory education tool (*n* = 1, 12.5%) and one (12.5%) availed of the opportunity to optimize their school food policy by booking an individual session with a member of the DAIRE research team. As this was low, all schools (100%) received a general document with advice relating to school food policies.

#### Engage intervention

With regards to Engage, 100% (*n* = 7) of Engage schools received intervention materials to enable complete intervention delivery. Notably, 100% (*n* = 7) of Engage schools implemented core delivery. However, as shown in Table 4, on average, Engage schools (n = 7) implemented 50% of the full (including core and optional Engage elements) intervention material, as outlined in Additional Files 8, 9 and 10.

**Table 4:** Summary of Engage elements implemented in schools

Engage schools (*n* = 7)	Completed 5 lessons with associated activities	Completed 1 talk from a guest speaker	Mean core minimum Engage delivered *n* (%)	Engage elements delivered per school (*n* = 26 in total) *n* (%)	Mean dose of Engage elements delivered in schools (%)	Mean (SD) number of Engage elements delivered in schools (*n* = 26)
C	✓	✓	7(100)	13(50)	50	13 (1.73)
D	✓	✓		12 (46.1)		
O	✓	✓		14(53.8)		
G	✓	✓		11(42.3)		
L	✓	✓		15(57.7)		
P	✓	✓		15(57.7)		
R	✓	✓		11(42.3)		

### Acceptability

Results of Likert-scale questions (Table 5) revealed high acceptability of the Nourish and Engage interventions by teachers within a NI primary school setting.

**Table 5:** Teacher Acceptability of the Nourish and Engage intervention as reported within the NEQ and ETEQ

	**Key Stage**	**Strongly Agree**	**Agree**	**Neither Agree nor Disagree**	**Disagree**	**Strongly Disagree**	**Mean Score (SD)**
**Part A) Nourish**							
***Q5**** Nourish resources, food items and activities useful and appropriate (n* = *16)*	N/A	14 (87.5)	2 (12.5)	0(0)	0(0)	0(0)	1.1(0.34)
***Q6**** Delivery of Nourish elements (snacks, canteen decorations) organised and easy to manage (n* = *16)*		9(56.3)	6(37.5)	1(6.3)	0(0)	0(0)	1.5(0.51)
***Q7**** Organisation of Tasting Days adequate (n* = *12)*		9 (75)	2(16.7)	1(8.3)	0(0)	0(0)	1.3(0.4)
***Q9**** Pupil reaction to the Nourish intervention elements (e.g. snacks. Canteen decorations, food presentation, Tasting Days) was generally positive (n* = *16)*		15 (93.8)	1 (6.3)	0(0)	0(0)	0(0)	1(0.25)
***Q10**** Nourish Tasting were a positive experience (n* = *14)*		11(78.6)	3(21.4)	0(0)	0(0)	0(0)	1.2(0.43)
**Part B) Engage**							
***Q4**** Engage Lesson plans useful and appropriate for year group (n* = *11)*	KS1 *n *= 9	3(33.3)	6(66.7)	0(0)	0(0)	0(0)	1.7(0.5)
	KS2 *n* = 2	1(50)	1(50)	0(0)	0(0)	0(0)	1.5(0.71)
***Q5**** Engage Guide timings within lesson plans used accurate and appropriate (n* = *10)*	KS1 *n* = 8	3(37.5)	4(50)	1(12.5)	0(0)	0(0)	1.8(0.71)
	KS2 *n* = 2	0(0)	2(100)	0(0)	0(0)	0(0)	2(0)
***Q7**** Available resources within the Farm to Fork, Pleasure on a Plate and Food Futures topics appropriate and useful for year group (n* = *10)*	KS1 *n* = 8	2(25)	6(75)	0(0)	0(0)	0(0)	1.8(0.46)
	KS2 *n* = 2	1(50)	1(50)	0(0)	0(0)	0(0)	1.5(0.71)
***Q9**** Pupil reaction to Engage lessons was positive (n* = *11)*	KS1 *n* = 9	6(66.7)	2(22.2)	1(11.1)	0(0)	0(0)	1.4(0.73)
	KS2 *n* = 2	1(50)	1(50)	0(0)	0(0)	0(0)	1.5(0.71)
***Q11**** Engage speakers or visits useful and appropriate (n* = *11)*	KS1 *n* = 9	5(55.6)	4(44.4)	0(0)	0(0)	0(0)	1.4(0.53)
	KS2 *n* = 2	2(100)	0(0)	0(0)	0(0)	0(0)	1(0)

Nourish data reported as n (%) of participant (n_max_ = 16) responses to the above statements

Engage data reported as n (%) of participant (nmax = 11) responses to the above statements

NEQ data completed by teaching staff, principal/vice principal or reception staff – i.e., school staff

Thematic analysis of Nourish and Engage qualitative data identified seven key themes and supports these findings as follows (Table 6).

**Table 6:** Thematic analysis of qualitative Nourish and Engage intervention data

**Themes**	**Codes**	**Illustrative Quotes**	**Formal and Informal (Study Records)**	
**Part A) Nourish**				
*Pupils enjoyed healthy snack provision*	• Children enjoyed snacks • Encouraged tasting of new foods which children had not tried previous • Hassle free prompt snack delivery • Easy access packaging of snacks • Sense of independence for children • Time-consuming to butter toast – but worth it	• “The children really enjoyed their snack this week. It might interest you to know that, for a few, it was their first time tasting wheaten bread! It all went down very well” [School E P7 teacher] • “Yes, the delivery took place promptly each Thursday and more than enough food for pupils provided” [School E principal] • “They loved having their bread toasted and the independence of buttering their own/preparing their own snack.” [School J teacher] • “When classroom assistant was there-easy to toast/butter etc. But very time consuming-lovely as a treat once a week” [School G teacher]	• DAIRE feedback and quotes document • Nourish Schools Field Notes • NEQ • NOT	• Formal n = 3 • Informal n = 3
*Well-organised and engaging tasting days*	• Well-organised • Children enjoyed and engaged • Encouraged to try new food items • Sense of responsibility • Age appropriate • All children’s diets catered for	• “Highly organized. Someone to meet us off the bus. Didn’t have to think of anything. Children were engaged throughout. Very worthwhile experience” [School G teaching staff] • “The experiences gave the children a chance to prepare food together e.g. making sandwiches. It gave them a sense of responsibility.” [School G teacher] • “Children shown video from FIP4, children all screamed ‘ahh’ when chicks were on screen. MP staff told children they had lots of different flavours of chicken with them today but that they ‘mixed them up’ so they needed pupils help to identify flavours. Children tried different flavours and had to guess e.g. plain chicken, BBQ, Sweet Chilli, Tikka Masala. Pupils very excited and shouted out answers/guesses for every flavour. Lots of hands up/interaction. Many pupils told me this was their favourite station” [DAIRE Research team member]	• DAIRE feedback and quotes document • Nourish Schools Field Notes • NEQ • NOT	• Formal n = 3 • Informal n = 3
*Useful canteen decorations*	• Easy and prompt delivery • Equipment and good addition to school	• “The equipment provided to the canteen has been most welcome and put to immediate use”. [School E principal] • “Delivery of canteen equipment was hassle free” [School E principal] • “… The canteen decorations will be an excellent addition to our canteen in the new school year and staff were delighted to receive them”. [School R teacher]	• NEQ • NOT	• Formal n = 2 • Informal n = 2
**Part B) Engage**				
*Enjoyable Engage topics, lessons and resources*	• Children enjoyed and engaged with interactive lessons and resources: videos, games, stories, songs, physical education lesson • Accessible resources • Age-appropriate	• “Age appropriate, pupils engaged and enjoyed, informative [School D P3 teacher] • “I liked that the resources were interactive and all links for online resources were easily accessible. The children enjoyed the story books and songs” [School O P3 teacher] • “The videos and lesson plans were very informative and provided great introductions for each area and generated good discussions too!” [School G P3 teacher] • “Fantastic enthusiasm-particularly Animal Welfare and Johnny Loves Milk. Children loved their PE lesson and stories from the Powerpoint” [School O P7 teacher] • “Lots of excitement, P3 kids very engaged and excited, salmon and jellyfish game, all pupils responded well” [DAIRE research team observing physical education lesson]	• Copy of perceptions PE class • Overview of Engage Delivery on Daire document • EOT • ETEQ	• Formal n = 2 • Informal n = 4
*Questionable age-appropriateness for P3 children*	• Attention span of younger children • Lessons too detailed and advanced	• “Some videos were a little long for P3 children. The ‘Right Royal Fishy Tale’ book was also a bit too long to hold my P3s attention throughout although they did love the storyline” [School R P3 teacher] • “The resources and activities were appropriate and useful…we found the Food Futures element harder to deliver as KS1 pupils aren’t as influenced by marketing etc.…some lessons needed to be simplified/adapted” – [School G P3 teacher]	• Tony Comments on DAIRE • Overview of Engage Delivery on Daire document • EOT • ETEQ	• Formal n = 2 • Informal n = 4
*Engaging guest speakers, trips and visits*	• Children enjoyed and engaged • Age-appropriate • Informative • Possible that money spent on trips was not worthwhile (*Note: only in one school*)	• “All speakers were knowledgeable on their subjects and engaged the children” [School C P7 teacher] • “Really engaged children-appropriate to age group. Great resources and follow up activities. Great to have a specialist to talk to children” [School G P3 teacher] • “The Farm was amazing. The children thoroughly loved it. The staff engaged so well with the children” [School D Principal] • “Address given incorrect..10 min visit…I just feel that the money spent on buses and the time spent travelling was a waste of funds which could have been spent elsewhere.” [School P Teacher]	• Overview of Engage Delivery on Daire document • ETEQ • EOT	• Formal = 2 • Informal = 3
**Part A Nourish and B) Engage**				
*Full Academic Year*	• Delayed DAIRE start • Rushed delivery • Plan throughout the year	• “There is a LOT of content. Need a whole school year to develop properly and do all lessons. The programme (Daire Project) began after Christmas so there was limited time to cover all topics, due to rest of curriculum having to be delivered.” [School P ‘Nourish and Engage’ P3 teacher] • “It was difficult to find time to organise/plan lessons-time constraints” [School G Teacher] • Due to time constraints and starting the programme during the year we were unable to deliver all the elements. [School L] • Limited lessons delivered due to time. We would love more term time to infiltrate into our planning throughout the year. [School O P3 Teacher]	• Nourish Schools Field Notes • NEQ • ETEQ	• Formal = 2 • Informal = 3

Within the Nourish intervention, Theme 1 was ‘Pupils enjoyment of the healthy snack provision’. Within the NEQ, teachers reported that healthy snack provision was the most valuable element of the Nourish intervention and that Nourish had a positive effect by encouraging children to try new food items, with even ‘fussy’ children consuming the snacks after observing their peers. Theme 2 identified within the Nourish intervention was ‘Well-organised and engaging Tasting Days’. Teachers reported that these days were well-organised, children were engaged and encouraged to try new foods and that this instilled a sense of responsibility. It was reported that this event was age-appropriate and all diets were catered for. Theme 3 identified within the Nourish intervention was ‘Useful canteen decorations’, as most teachers reported that delivery of canteen decorations (e.g. posters, bunting and tablecloths) was prompt and effortless and decorations were a useful addition to the school.

Within the Engage intervention, 4 themes were identified. Theme 4 was ‘Enjoyable Engage topics, lessons and resources’. Teachers reported that interactive lessons and resources such as videos, games and the practical physical activity (Physical Education) lesson were the most popular aspects of Engage and that the *Animal Welfare* and *Johnny Loves Milk* lessons were particularly favoured. Theme 5 identified within the Engage intervention was ‘Questionable age-appropriateness for P3 children’ whereby some concerns were raised in relation to lessons being too advanced and detailed when considering the attention span of younger children. With respect to the Engage Theme 6 of, ‘Engaging guest speakers, trips and visits’, teachers emphasized that children enjoyed these Engage elements, finding them extremely informative and useful. The final theme consistently identified within both Nourish and Engage qualitative data was that the DAIRE intervention should be implemented across a full academic year. Most teachers commented on timing and planning pressures and this, combined with the fact that DAIRE did not begin until late into the second academic term, meant that delivery was rushed.

“There is a LOT of content. Need a whole school year to develop properly and do all lessons” [School P ‘Nourish and Engage’ P3 teacher]

### Contamination

No formal data were collected in relation to contamination but it was possible to make some assumptions based on consideration of the likelihood of DAIRE control group participants being exposed to the Nourish and Engage interventions through examining the DAIRE intervention study design, recruitment, randomisation and allocation. Based on this information, it is unlikely that contamination influenced the intervention and outcome data collection as a result of study design and randomization (Additional File 11).

### Mechanisms of Impact

As interviews with pupils were not conducted, it was not possible to explore potential mediators of behaviour change or unanticipated pathways within this process evaluation. However, thematic analysis of qualitative teacher responses within the NEQ and ETEQ allowed insight into pupil reaction and engagement with the interventions and exploration of possible DAIRE MOI.Two possible MOIs in relation to the Nourish intervention were identified (Table 7):

1. Social learning
2. Experimental Learning (hands-on approach)

**Table 7:** Mechanisms of change uncovered from NEQ and ETEQ data

**Mechanism of impact**	**How it worked**	**Illustrative Quotes**
**Part A) Nourish**		
*Social Learning*	• Peer influence • Pupils imitate pupils • Class discussions • Preparing food together	• “When given the wheaten bread/brown bread first a number of the children had never had before and didn’t try it but eventually most of them were eating it and really enjoying it. As often as possible we had the snack along with the P7-great peer tutoring opportunities.” [School L teacher] • “The children became more independent at preparing their snacks, helping each other etc. and looked forward to the days snacks” [School J teaching staff] • “The children looked forward to their snacks; even tasting wheaten bread for the first time and enjoying it! They enjoyed having toast in the classroom.. They shared out their fruit with each other if they didn’t fancy eating some of them.” [School G teacher]
*Play-based learning*	• Increased knowledge of healthy food • Sense of independence and responsibility • Children involved in decision making	• “The healthy snacks encouraged healthy eating. The Tasting Day taught the children a lot and again encouraged healthy eating.” [School R teacher] • “Children loved having the choice of their own snack. They loved having their bread toasted and the independence of preparing their own snack.” [School J teaching staff] • “The experiences gave the children a chance to prepare food together e.g. making sandwiches. It gave them a sense of responsibility.” [School G teacher – Regarding Tasting Day at C1]
**Part B) Engage**		
*Interactive engage content*	• Class discussions • Group work • Interactive lessons – stories, songs, videos	• “Really engaged children-appropriate to age group. Great resources and follow up activities. Great to have a specialist to talk to children.” [School G P3 teacher – Regarding guest speakers] • “Children loved planning their own menus and enjoyed presenting their ideas to the class. Lessons allowed for lots of group work and discussion. Everyone was included – re Food Futures: Restaurant lesson” [School L P3 teacher] • “Fantastic enthusiasm-particularly Animal Welfare and Johnny Loves Milk. Children loved discussions/debates. Children loved their PE lesson and stories from the PowerPoint” [School O P7 teacher]
*Real life connections*	• Relate to real life scenarios • Brought DAIRE to life • Memorable lessons and visits	• “Children really liked making their own book as part of the ‘Food Scribblers’ lesson. They are enjoying the Food Futures lessons and are excited to cook the meal chosen by the class. The visit of the travelling farm was one of their favourite parts” [School R P3 teacher] • “Some children were able to contribute real life experiences due to family farm links” [School L P3 teacher] • “The children loved the Animal Welfare lesson and related it to how they look after their pets at home!” [School R P3 teacher]

Most teachers reported that social learning and peer influence was key to healthy snack consumption, with pupils being more willing to try new foods and opting to consume the Nourish healthy snacks once they observed their peers doing so.

The Nourish intervention is also likely to have produced change by increasing pupils’ knowledge about food and healthy food options through play-based learning and experiences.

MOI for Engage were explored as, although no statistical differences were observed regarding primary outcomes, teachers reported the benefits of Engage and commented on how it should be integrated into the school curriculum.

Thematic analysis identified two possible MOI in relation to the Engage intervention (Table 7):

1. Interactive engage content
2. Real life connections

Teachers commented that the Engage topic and lesson content was interactive, stimulating and engaging for children and encouraged class discussions. The Engage intervention also allowed pupils to establish real life connections. Relating lessons to real life i.e., *Animal Welfare* lesson to the children’s pets, cooking a meal together, attending the pet farm and listening to guest speakers brought the Engage topic to life.

### Context

Teachers from 50% (*n* = 4) of Nourish schools and teachers from 71.4% (*n* = 5) of Engage schools reported experiencing barriers during intervention delivery. Thematic analysis of NEQ and ETEQ qualitative responses revealed that time was the biggest DAIRE barrier which, in turn is likely to have influenced the time to induce pupil change and, ultimately, the change in primary and secondary outcomes observed. Teachers had to give priority to delivering the national curriculum and other school priorities and, as such, they reported shortening lessons or not completing lessons due to time constraints:

“Time was a major issue. With everything else needing to be taught it was difficult to fit it in” [School C Engage P3 teacher]

Teachers also reported that DAIRE should be planned well in advance of the school year to enable complete implementation. Overall PE findings detail the main barrier to DAIRE intervention implementation was time, which in turn was likely to have influenced the time to induce pupil change and, ultimately, the change in primary outcomes observed.

However, findings on fidelity and dose, acceptability and MOI, suggested that guest speakers, the Mobile Petting Farm, healthy snack provision and Tasting Days were the most popular elements and the ones that had a notable positive influence.

Half (*n* = 5, 50%) of Nourish schools and 42.9% (*n* = 3) of Engage schools reported that other similar health initiatives were in place whilst the Nourish intervention was being delivered. Of these, 28.6% (*n* = 2) Engage schools reported that a ‘Healthy Eating and Fitness week’ took place whilst implementing the Engage intervention.

In total *n* = 3 Nourish schools (20%) and *n* = 1 Engage school (14.3%) reported that a substitute teacher had been in place for a significant period of time during intervention delivery. Of these schools, 100% (*n* = 4) of teachers were provided with a project DAIRE handover and were therefore aware of how to implement the intervention appropriately.

It is also important to reiterate that in terms of barriers, there was no cost to schools in relation to their involvement in DAIRE.

The DAIRE trial originally planned to incorporate a parent/guardian aspect within the DAIRE interventions as much of the evidence supports the involvement of parent/guardians in such interventions [8]. However, due to time constraints, the complexity of the trial and capacity within schools, this was not pursued and included in the final interventions.

## Discussion

DAIRE findings reported by Brennan et al., [8] indicated that the Nourish food environment intervention produced more positive changes in emotional and behavioural wellbeing, food knowledge, cooking competence and dietary intake than the Engage food-education intervention in primary school children in an economically disadvantaged area in Northern Ireland. The aim of this study was to conduct a process evaluation to help evaluate implementation of the Nourish and Engage interventions, the mechanisms of impact, the context and consequently elucidate why the Nourish intervention was more effective than the Engage intervention and inform any modifications for successful future UK-wide DAIRE rollout.

DAIRE had a 100% retention rate at both school and pupil-level which appears higher than observed in other school-based dietary interventions [17, 18], although retention rates are often not reported in school-based diet/food environment process evaluations. Process evaluation results, along with teachers’ follow-up feedback, indicated high levels of acceptability and engagement with both the Nourish and Engage interventions and these two factors have previously been identified as integral to intervention success [19–21]. Methods used to determine acceptability and contamination vary widely across the PE literature, so it is difficult to directly compare these elements; however DAIRE acceptability and risk of contamination also appears comparable with other PE literature with no issues identified for either element.

Findings on quality (fidelity) and quantity (dose) of DAIRE implementation revealed that Nourish schools implemented a higher mean intervention dose in comparison with Engage schools which may help explain its effectiveness when compared with Engage in terms of the outcomes. It was notable that 100% of schools randomised to Engage delivered core elements but mean delivery of all Engage intervention content available to schools was only 50%, largely due to teacher time pressures. DAIRE trial results found that the Engage intervention appeared to be less effective than the Nourish intervention so the poorer implementation of Engage may help explain this. However, this PE yielded valuable information in relation to the time pressures experienced by teachers and the feedback received in relation to the excessive level of Engage content and must be taken into account in future trials or DAIRE roll-out, in order to ensure optimum delivery and effective implementation. A delayed DAIRE intervention start date, in March rather than February 2019, resulted in late delivery of some Nourish intervention elements (May 2019) and the closure of schools for the summer recess in June 2019, is likely to have affected Nourish implementation and, as a result, observed primary and secondary outcomes. However, DAIRE implementation levels are comparable with those reported within other school-based interventions targeting fruit and vegetable consumption, physical activity, dietary behaviour and obesity prevention (range of 30% to 97% reported) [17, 18, 22–26].

Although teachers reported making adaptations to both interventions, largely to reduce amount of content due to time pressures, it is difficult to get a sense of the extent of adaptations from the data collected. This is notable as Inchley et al., [27] reported that teachers feeling empowered to adapt interventions can enhance intervention effectiveness. In contrast, however, adaptations to Engage content in terms of lesson reduction may have influenced fidelity and could possibly have contributed to the lack of change produced in those randomised to the Engage intervention. It was not possible to consider fidelity of training materials given to teachers as part of DAIRE but would be of interest to explore this further in future in terms of more comprehensive teacher training in relation to intervention delivery.

PE results revealed the intervention elements most implemented required the least teacher effort. These results are in-line with process evaluation results of the Child and Adolescent Trial for Cardiovascular Health and High 5 school-based interventions, whereby more time-consuming activities were implemented less frequently by teachers in the classroom [26, 28]. A systematic review of the implementation of 24 school-based fruit and vegetable distribution interventions highlights the need for detailed implementation guidelines and training workshops for teachers to ensure high intervention fidelity [19, 29]. The Engage resources in DAIRE included a video aimed at teachers, but more detailed guidance/training, alongside more classroom planning time are likely to be of value.

There were also varying levels of teacher engagement in relation to outcome assessment, although this is distinct from the intervention delivery. In contrast, PE findings regarding high pupil questionnaire response rate demonstrate the high level of engagement in relation to data collection in children which will be encouraging in relation to any future DAIRE rollout. Ultimately, stronger teacher engagement with outcomes assessment could have resulted in more reliable conclusions regarding DAIRE outcomes. Educational attainment data were also requested from participating schools but the limited data received from a small number of schools (n = 5) were variable in format and did not allow for formal analysis, which highlights the need for standardisation of data collection methods in any future evaluation.

Four possible MOI: social learning and play-based learning in relation to Nourish, interactive engaging content, and real-life connections in relation to Engage, were identified from process evaluation findings. Fidelity, dose and acceptability results indicated that Nourish healthy snacks and Tasting Days, and Engage lessons involving story books, songs and process evaluation lessons, as well as Guest Speakers and the Mobile Petting Farm were the key DAIRE intervention elements. The majority of these identified key elements were delivered as part of the Nourish intervention specifically, which may explain its effectiveness compared with Engage in relation to the trial outcomes. The Nourish Tasting Days and healthy snack provision may have produced the significant changes observed in the DAIRE trial in terms of increased willingness to try new foods through pupil bonding and shared learning. Teachers commented in the NEQ that preparing healthy snacks and feeding back product preferences during Tasting Days influenced childrens’ awareness of healthy food options and helped instil a sense of responsibility and independence in children regarding their food intake and dietary choices. Such elements would be costly, therefore the removal of the cost barrier to DAIRE may be considered a facilitating factor. These findings are notable, as such factors must be retained and replicated in future delivery.

These findings are comparable to PE findings from other school-based interventions including Project Tomato and High 5, whereby the most popular pupil activities were interactive [28, 30]. In terms of ensuring intervention effectiveness and successful future UK-wide rollout, PE findings suggest that an overall focus should be placed on retaining the key MOI, and ensuring DAIRE is interactive and easy to implement for teachers.

It is evident from PE results that lack of time was a key contextual barrier influencing DAIRE implementation and was most reported in relation to Engage, which relied more on teacher delivery. This finding is consistent with PE findings from Christian et al. [30] regarding Project Tomato (n = 311 children aged 7 years in 24 schools) and Bere et al. [31] regarding Fruit and Vegetables Make the Marks (n = 369 children, mean age 11.9 years, in 9 schools); whereby low implementation levels and little difference in children’s dietary intake was observed in schools as a result of intervention materials requiring too much preparation time. Process evaluation identified that teachers felt that DAIRE should be implemented across the full academic year and be integrated into the school curriculum. Similarly, previous PE studies of the We Act and the Active for Life Year 5 primary school-based interventions, reported integration of intervention content into the curriculum as a possibility to improve implementation and outcomes [20, 32]. DAIRE Engage intervention content was mapped to the NI curriculum, but as already discussed, planning time for teachers is likely to have been limited so this should be built into any further rollout.

Half of Nourish schools and 42.9% of Engage schools reported that other similar health initiatives were in place whilst the Nourish intervention was being delivered; however, the 11 week time-frame over which Engage was implemented vs the 5 days associated with similar health initiatives and the minimal number of schools that took part in this, resulted in this being of minimal importance. Furthermore, for both interventions, data at baseline and end-point accounted for the healthy eating policy in place in schools, ensuring that results observed are solely attributed to the intervention. Overall, a relatively small percentage of both Nourish and Engage schools reported that a substitute teacher had been in place for a significant period of time during intervention delivery. Of these schools 100% (n = 4) of teachers were provided with a project DAIRE handover and were therefore aware of how to implement the intervention appropriately; therefore it is likely there was no significant impact on intervention delivery.

Originally it was planned for Nourish to include more of a focus on canteens and food served rather than food presentation, however, procurement regulations within schools and the need to adhere to food standards precluded this within the timeframe. It is likely that influencing procurement chains within school systems would be challenging as contracts are typically already in place so it is important to be aware of these potential challenges when designing future studies. Catering staff feedback suggested issues with limited time to prepare food and time allocated for pupils to eat the food, therefore these considerations need to be built into any rollout of the programme.

The use of the socio-ecological model (SEM) [33] was not formally considered during the Nourish or Engage intervention development. However, on reflection, DAIRE interventions took into consideration the importance of the school environment and the networks of people and structures that surround pupils’ experience of school life. The Nourish intervention targeted the individual level through healthy snack provision, encouraging individual choice of healthy and local foods and cookery activities; the interpersonal level through cookery activities, group learning, sensory education and chef demonstrations; the organizational and environmental levels through enhancing the dining and physical experience through provision of canteen equipment, food provision for school event, themed food days and school food policy recommendations. The Engage intervention targeted the individual level through lessons which included personal feedback, demonstration of behaviour and self-monitoring, the interpersonal level through group learning activities and the organizational level through integrating Engage into the curriculum. The extent of the SEM levels involved in the DAIRE interventions may also help explain main trial results as Nourish more extensively involved each level of the SEM and was found to be more effective than the Engage intervention overall. The effectiveness of multi-level SEM involvement in interventions such as these was reported in a systematic review of interventions which aimed to decrease sedentary time in children [34]. In this systematic review, it was reported that interventions tended to be more effective when they involved 4 levels, using both agentic and structural strategies and the relevance of multilevel strategies to reduce sedentary lifestyle in children was highlighted [34]. A multilevel SEM school-based approach promoting physical activity also demonstrated effectiveness in relation to weight change beyond 2.5 years after the intervention ended and it was thought that the multilevel approach involving communities and families better supported the integration of physical activity in everyday life and helped achieve long-term changes [35]. A study which developed and applied a SEM for healthy eating in schools reported that school approaches to food should involve both organizational and community aspects of the school, implementing policies and rules towards food and influencing the social networks within the school and between schools and the wider community [36]. As such, the consideration of the SEM in taking forward the Nourish intervention may therefore be of value.

The research aimed to conduct with trial with ecological validity, where possible, but it was not feasible in terms of time and funding to translate all Engage intervention resources in the event Irish language schools were randomized to the Engage arm of the trial and this may be an important consideration in any future roll-out in regions where different languages are necessary. However, in order to achieve a whole-school approach and its prospective benefits to children’s diet quality and nutritional knowledge, the Engage education intervention should be considered for retention in any future DAIRE rollout. This may induce a sense of community amongst pupils, ensure consistency in the healthy eating messages both within and outside of the classroom; key here is making the lessons fun and memorable for participating children, whilst also extending their knowledge.

Follow up questionnaire responses were generally very positive and supported the main PE results; highlighting particularly beneficial aspects, including the popularity of the healthy snack provision, enhancement of healthy eating polices and introduction of a sensory eating component. They also identified time constraints as a barrier to implementation.

This process evaluation has several strengths. It followed the recently updated MRC guidance and employed a comprehensive approach to evaluate all three framework elements. Due to prior planning, process-evaluation-specific tools and questionnaires were developed and used throughout the DAIRE intervention. Also, due to the large volume of DAIRE PE data collected, merging of data sources occurred, after discussion with the research team, to enable the most effective interpretation and use of data collected in terms of data quality and relevance for each aspect of process evaluation.

Primary schools in regions of socio-economic disadvantage in the North-West of Northern Ireland were targeted during recruitment for Project DAIRE. However, it is likely that results from the trial and this PE are generalisable to primary schools across Northern Ireland, and potentially culturally adapted for use across the UK and beyond further afield to beyond remainder of the UK and Republic of Ireland, as schools from areas of varying socio-economic status were recruited from both urban and rural locations. A number of factors reduced the likelihood of contamination of control participants in this trial, including whole school rather than class randomization and adequate training and experience of the DAIRE research team when carrying out data collection in control schools. Therefore, it is unlikely that contamination influenced any of the DAIRE primary or secondary outcomes observed (Additional File 10).

In terms of limitations, data collected from teachers that completed the PE questionnaires may not be representative of all teachers who were involved in the implementation of DAIRE. Furthermore, there is potential for bias in data collection as some of the researchers involved in main study outcome data collection were involved in PE data collection. The researcher leading the initial PE analysis was not involved in the DAIRE trial in any way, but researchers involved in the trial reviewed and commented on the final PE manuscript. Additionally, no direct pupil feedback regarding the DAIRE intervention exists. A poor response was received from schools in relation to the observations planned using the NOT and EOT tools to be completed by study researchers. It is likely that this hesitancy from schools is likely to be related to time pressures reported by teachers in this PE and the high level of content teachers were delivering as part of DAIRE. As such, this will be important to take this into consideration in any future roll-out. Whilst it would have been interesting to look at level of implementation or other outcomes by individual schools’ socio-economic status, the limited data we had on this aspect did not allow further analysis. Planned follow-up teacher phone calls and interviews were also not possible due to the COVID-19 pandemic, therefore no data on DAIRE intervention sustainability were collected and triangulation of DAIRE PE results was not possible. In addition to this, the majority of school principals did not respond to requests for the DAIRE research team to attend to conduct Nourish and Engage PE-related observations, therefore observations were limited. In relation to researcher positionality in the context of the qualitative element of the work, the main researcher was a postgraduate student researcher familiar with the education system in schools across Northern Ireland as a result of living within the wider province but does not have experience as a teacher or pupil at these specific schools or this specific socio-demographic region. The main researcher recognised that their identity and experience may impact data analysis and interpretation, so the wider research team were involved in all aspects of coding and identification and interpretation of themes to ensure this aspect of the work was conducted appropriately. Due to the limited nature of the qualitative data available for this PE and the fact that it was not possible to conduct pupil or teacher-facing interviews or focus groups, it was not possible for the research team to design the qualitative aspect with due consideration given towards our epistemological stance. Any future implementation of DAIRE would benefit from a more formal and extensive qualitative element:

Within this PE, data on mediating and moderating factors were not collected. Within the MRC framework [9], Mechanisms of Impact can incorporate mediational analysis of quantitative mediators and Context can incorporate quantitative testing of hypothesised moderators. If these had been measured we may have been able to perform a more detailed analysis in this regard which may have helped elucidate findings further in relation to why health-related quality of life significantly improved in males randomised to Nourish significantly compared with those who did not receive Nourish in subgroup analyses [8]. Therefore, future work exploring mediating and moderating factors may be informative in terms of interpreting these findings. Finally, PE was completed following the main DAIRE study outcomes being published; thus biased interpretation is possible. However, as initial PE analysis was undertaken independently by a researcher who was not directly involved with the DAIRE implementation and in-person data collection, the likelihood of this is reduced.

Gaps for future DAIRE research identified from this PE include: sourcing a sustainable funding and resourcing mechanism for future rollout, including a range of funding models and the role of the private sector. The research team had originally intended to conduct follow-up stakeholder interviews but due to the onset of the COVID pandemic it was not possible to complete these. It would also be of interest to conduct more formal and extensive qualitative work with pupils themselves in relation to behaviour change in the form of semi-structured interviews or focus groups which may further elucidate the most effective elements of such interventions. Incorporation of a ‘family’ and/or community aspect to the interventions to fully achieve a multi-level whole-school approach should also be considered. Overall, it is likely that DAIRE intervention success is generalisable to UK-wide primary schools with consideration given towards any adaptations to reflect cultural diversity and beyond, due to the comparable structures within the primary school education system, therefore PE findings can help to inform the plan and design of future similar effective interventions. Furthermore, if gaps in the research are addressed and the suggestions and considerations that are identified through this PE are implemented in a future roll-out, it is likely the improvements would enable easier implementation in the primary school setting and improve sustainability.

In conclusion, the DAIRE PE yielded several important findings related to the implementation, MOI and contextual factors influencing DAIRE intervention effectiveness and outcomes and helped to elucidate why Nourish produced more positive changes in outcomes than the Engage intervention. The most effective elements of the interventions which should be maintained in any future rollout include provision of interactive and engaging intervention elements at no financial cost to the school. Findings also identified suggestions for improvement including provision of increased teacher training, support and planning time, content reduction to facilitate easy integration into the existing school curriculum, and implementation across the full academic year.

## Supplementary Information

Below is the link to the electronic supplementary material.

Supplementary file 1.

Supplementary file 2.

Supplementary file 3.

Supplementary file 4.

Supplementary file 5.

Supplementary file 6.

Supplementary file 7.

Supplementary file 8.

Supplementary file 9.

Supplementary file 10.

Supplementary file 11.

## Data Availability

No datasets were generated or analysed during the current study.

## Abbreviations

MOI: Mechanisms of impact
MRC: Medical Research Council
HRQOL: Health-related Quality of Life
NEQ: Nourish Evaluation Questionnaire
ETEQ: Engage Teacher Evaluation Questionnaire
NOT: Nourish Observation Tool
EOT: Engage Observation Tool
SDQ: Strengths and Difficulties Questionnaire
KS: Key Stage
