# Supplementary figures and images for: Process evaluation of Project Daire: a food environment intervention that impacted food knowledge, wellbeing and dietary habits of primary school children
Source: BMC Public Health. 2025 Feb 6;25:486. doi: 10.1186/s12889-025-21628-4 (PMC11800617; doi:10.1186/s12889-025-21628-4)

**Additional File 1: The specific components of the DAIRE interventions**

**
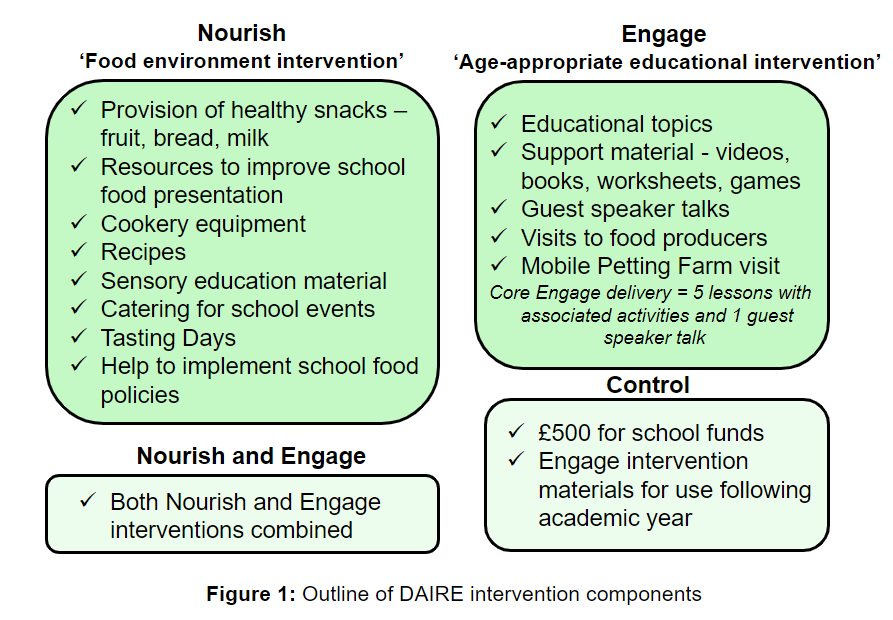
**

Supplement: Supplementary file 1 [file 12889_2025_21628_MOESM1_ESM.docx]

**Additional File 2: Nourish Evaluation Questionnaire**


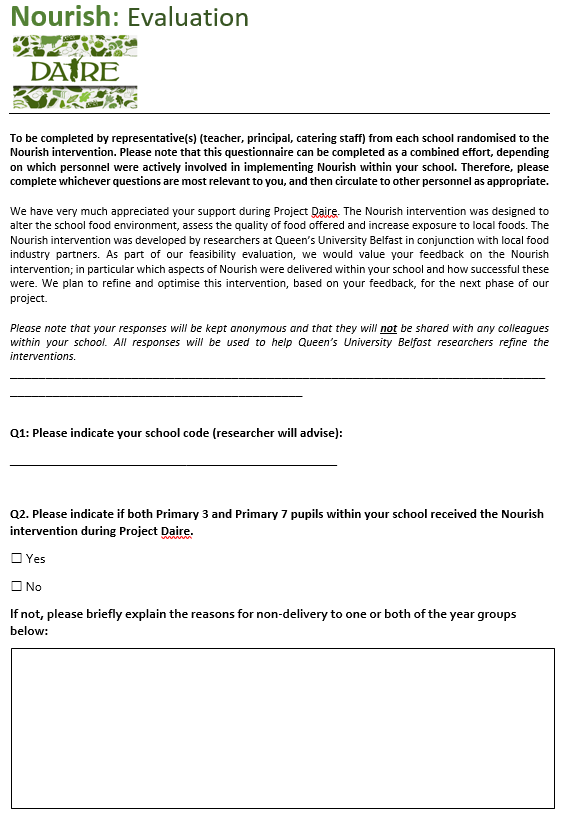


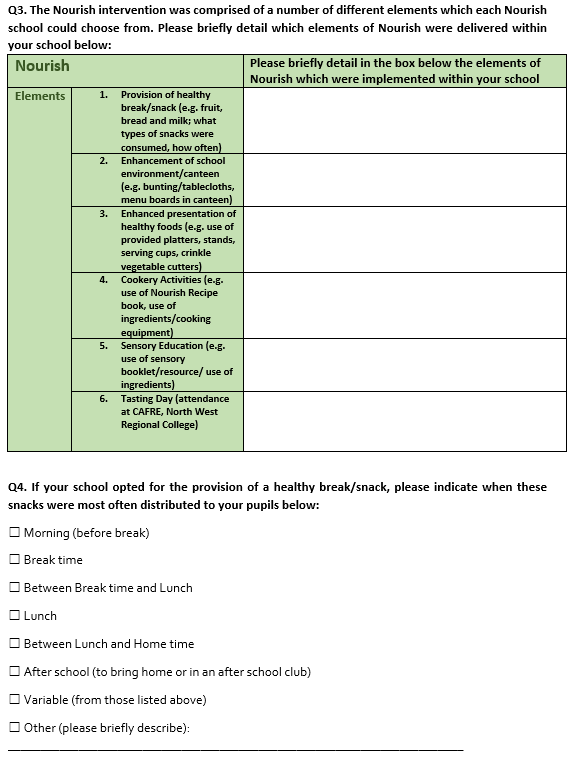


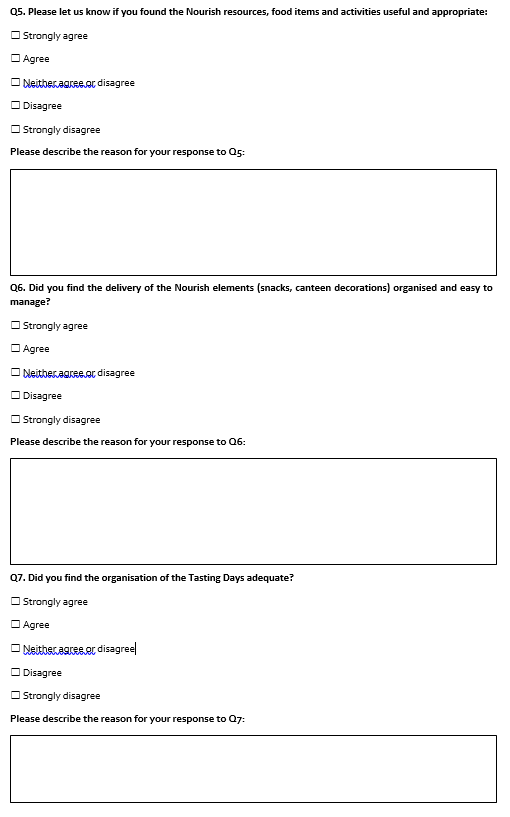


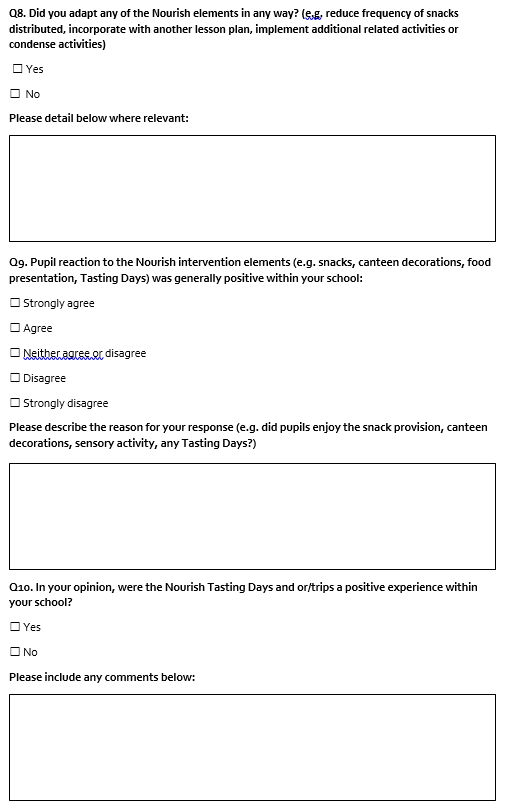


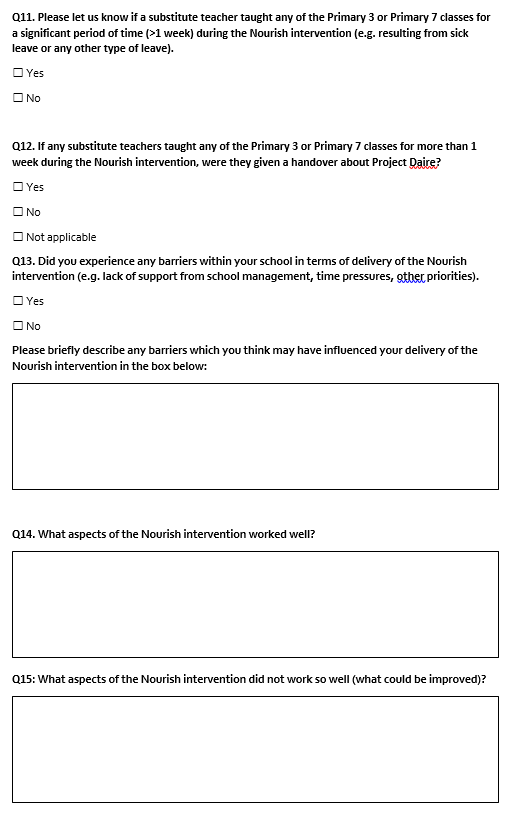


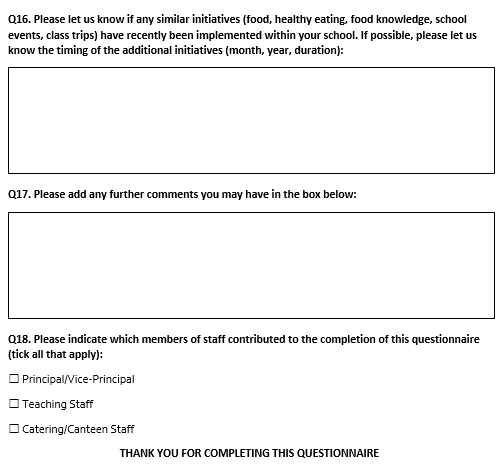

Supplement: Supplementary file 2 [file 12889_2025_21628_MOESM2_ESM.docx]

**Additional File 3: Engage Teacher Evaluation Questionnaire**


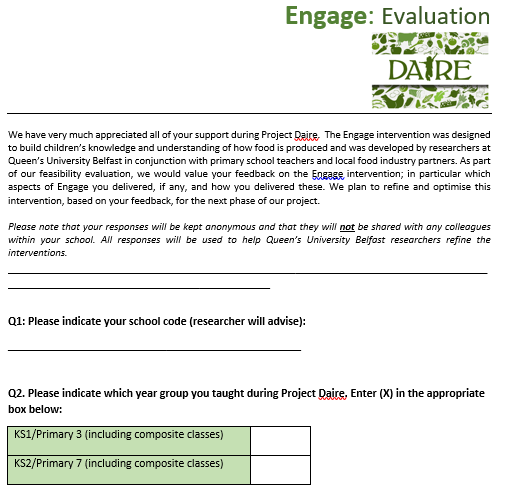


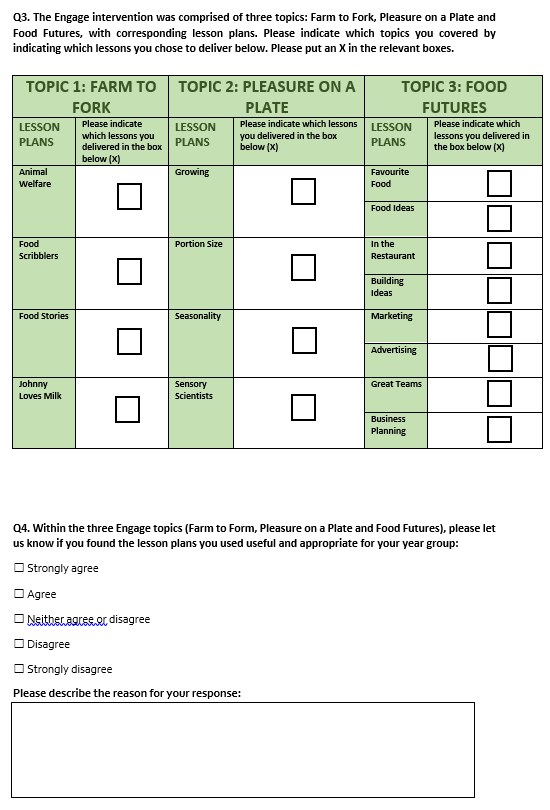


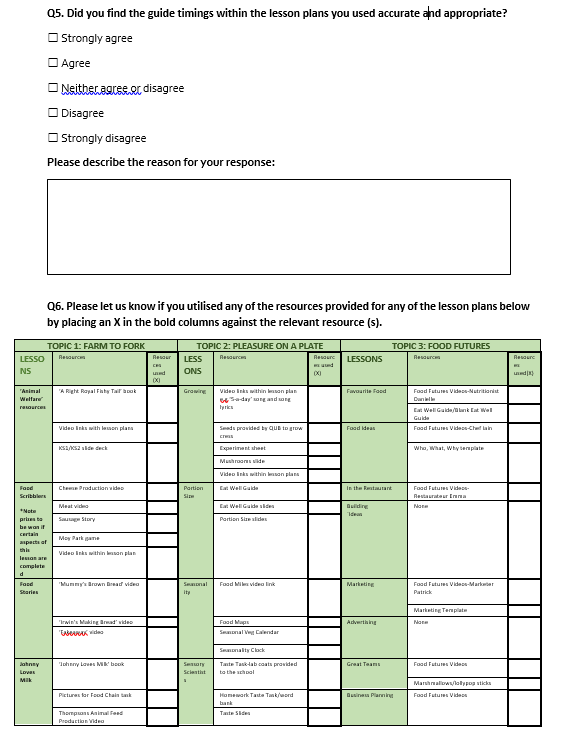


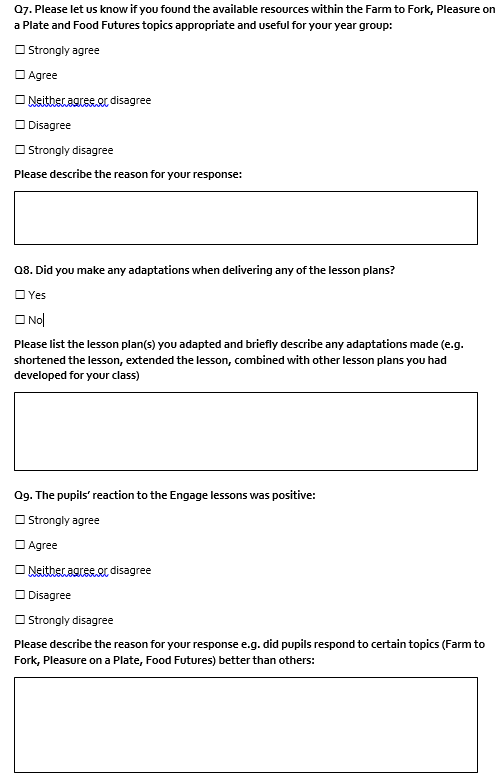


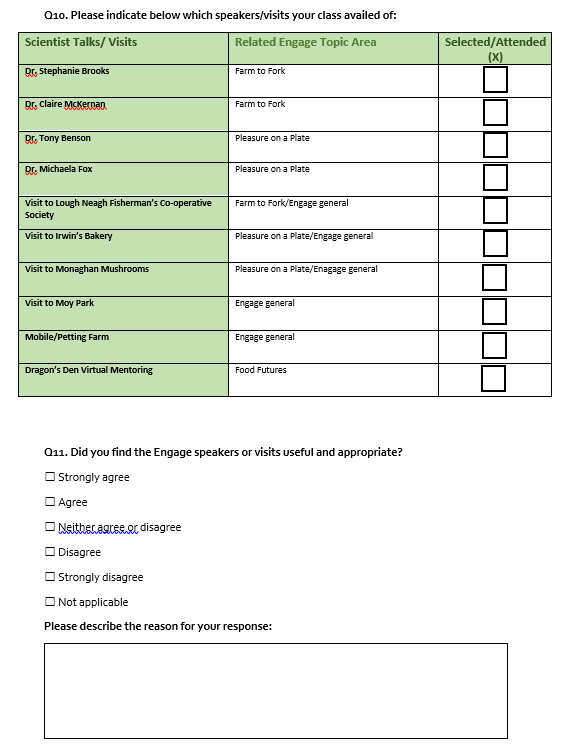


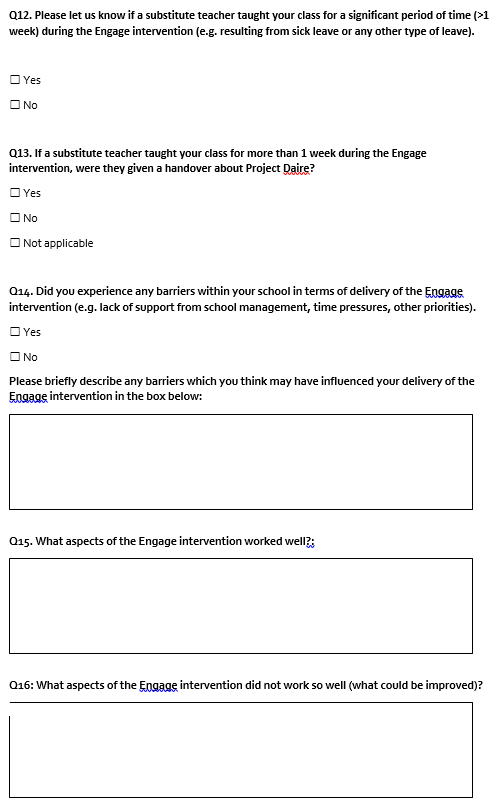


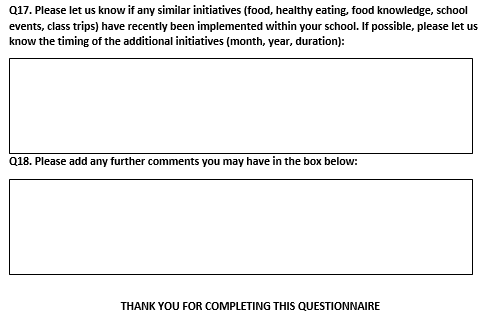

Supplement: Supplementary file 3 [file 12889_2025_21628_MOESM3_ESM.docx]

**Additional File 4: Nourish Observation Tool**

**
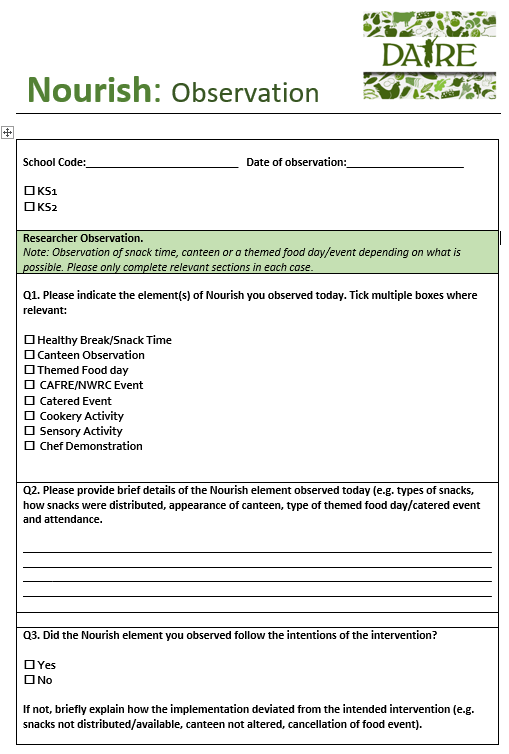
**

**
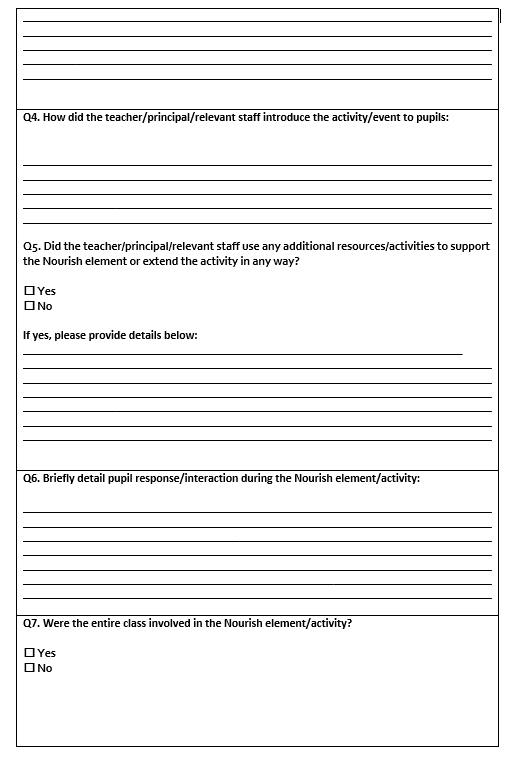
**

**
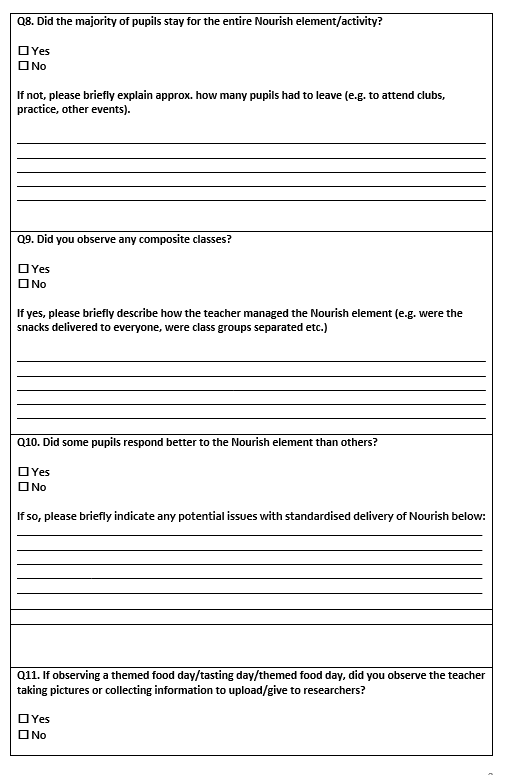
**

**
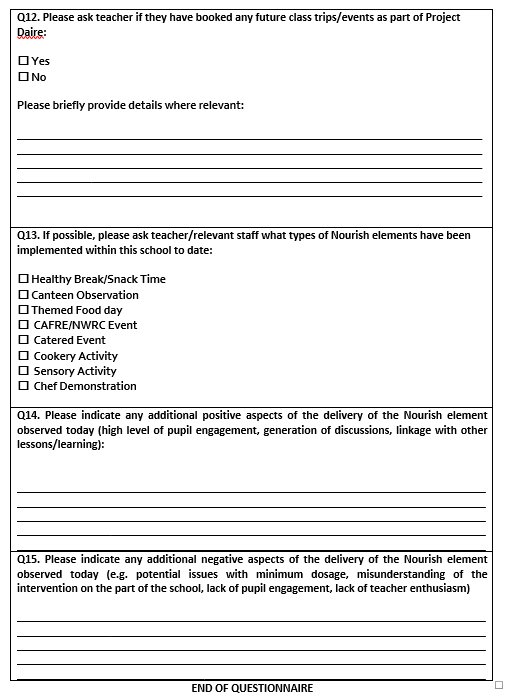
**

Supplement: Supplementary file 4 [file 12889_2025_21628_MOESM4_ESM.docx]

**Additional File 5: Engage Observation Tool**

**
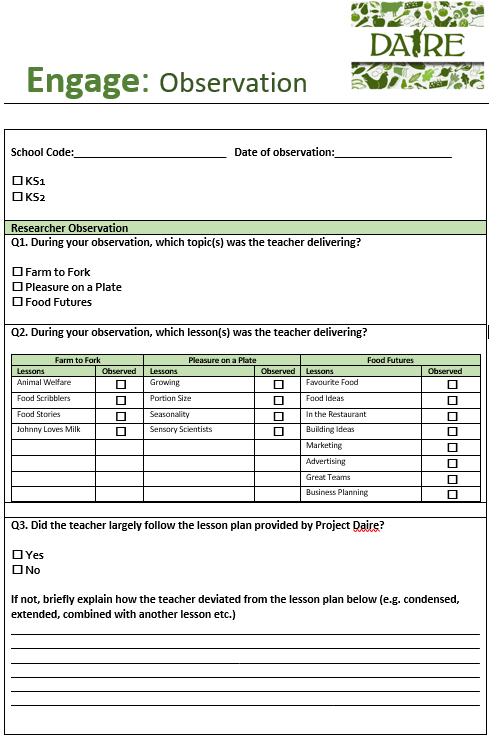
**

**
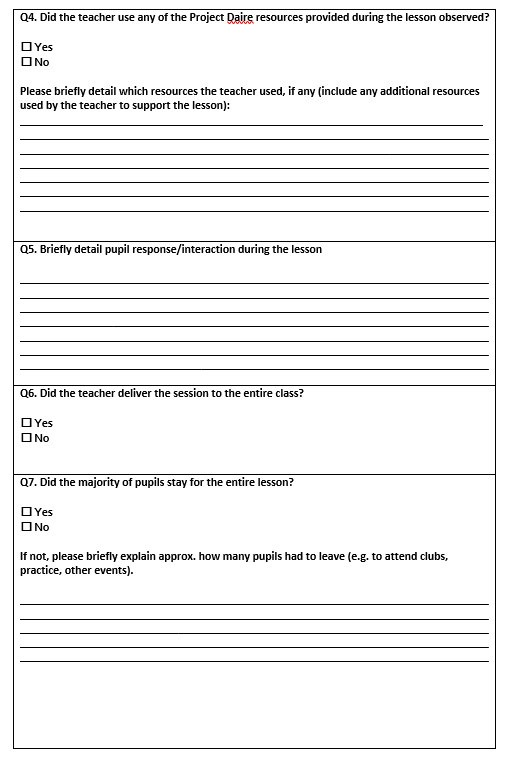
**

**
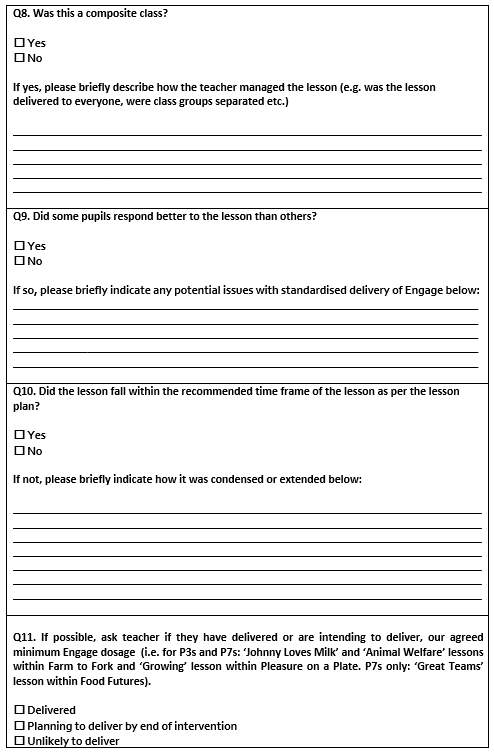
**

**
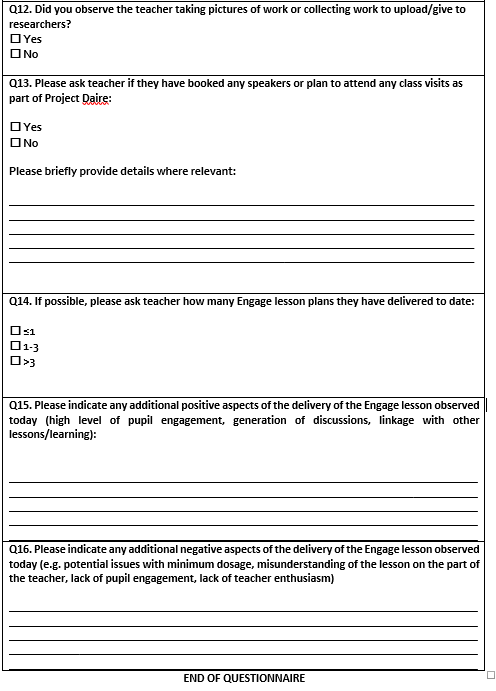
**

Supplement: Supplementary file 5 [file 12889_2025_21628_MOESM5_ESM.docx]
