# Supplementary material for: Process evaluation of Project Daire: a food environment intervention that impacted food knowledge, wellbeing and dietary habits of primary school children
Source: BMC Public Health. 2025 Feb 6;25:486. doi: 10.1186/s12889-025-21628-4 (PMC11800617; doi:10.1186/s12889-025-21628-4)
Supplement: Supplementary file 6 [file 12889_2025_21628_MOESM6_ESM.docx]

**Additional File 6. Follow-Up Questionnaires.**

| **Project DAIRE: Nourish and Engage Intervention: School Principal** |
| --- |

**Improving primary school children’s knowledge of and interest in food**

The ‘Nourish’ arm of the intervention was based on a food intervention to enhance the current school food environment of participating schools and to increase exposure to Northern Ireland-sourced food. The ‘Nourish’ intervention involved provision of healthy snacks which were provided during the school day and resources were also given to improve the school food environment and school food presentation. The ‘Nourish’ intervention incorporated, tasting Days, cookery equipment and recipes, sensory education and catering for school events.

The ‘Engage’ arm of intervention was an educational intervention promoting food, agriculture, science and careers associated with the current curriculum in Northern Ireland. The intervention was aimed at P3 and P7 children with lesson plans and classroom activities delivered by teachers, guest speakers and a visit to the farm were also offered.

The ‘Engage’ intervention was based on three topic areas;

Topic 1: Farm to Fork: To increase understanding of where food comes from, how it is grown or produced and how it reaches our plate.

Topic 2: Pleasure on a plate: To enable children to learn about the foods that make up a balanced diet and to increase children’s awareness of buying, preparing and eating food.

Topic 3: Food futures: To enable children to learn about how food production is changing and how new products are being developed.

Activities were developed by the research team and a range of stakeholders to support the lesson plans which included videos, worksheets, games, talks/visits from experts, visits to industry partner workplaces, class trips and practical experiments.

Resources

Videos (Rearing farm, Breeding farm, The hatchery and The Broiler farm, meat video, cheese production, making bread, animal feed), Books (The Right Royal Fishy Tale, sausage story, Johnny love’s milk), games (Say what you see) and worksheets (Eatwell guide, advert templates, who, what why) songs. Sensory education resources, use of recipe books, cookery activities.

**The survey will take approximately 10-15 minutes to complete.**

Top of Form

**Question Title**

**1. Could you please explain your overall experience of project DAIRE?**

**Question Title**

**2. Do you have a school food policy in your school? And do you feel that project DAIRE has supported this? Have you made any changes to your school food policy, or have you any plans to make changes to your school food policy, since project DAIRE?**

**Question Title**

**3. Since project DAIRE ended in June 2019, are you aware of any components of DAIRE being replicated in this academic year? (e.g. Topic areas, lesson plans, activities, healthy snack provision, school canteen resources including; table clothes, posters, menu boards, presentation of school food or cookery activities including; cookery equipment, recipe books).**

**Question Title**

**4. Are there any parts of DAIRE which could not be used last year due to time constraints, which are now being used in this academic year? (e.g. Topic areas, lesson plans, activities, healthy snack provision, school canteen resources including; table clothes, posters, menu boards, presentation of school food or cookery activities including; cookery equipment, recipe books).**

**Question Title**

**5. Since project DAIRE ended in June 2019 has there been any new science or food related initiatives introduced in your school and explain your reasons for these? (e.g. Breakfast clubs).**

**Question Title**

**6. What are your thoughts on healthy snack provision? (e.g Impact, benefits, implications, best time of day).**

**Question Title**

**7. If DAIRE was to be implemented again in your school what parts of the intervention would you utilise the most and why? And is there anything that might help your school implement DAIRE into your school? (e.g. further training, support from researchers).**

**Question Title**

**8. Do you think it would be useful if the research team delivered an introductory Project DAIRE information session to all teachers and caterers involved in DAIRE prior to the intervention start date?**

**Question Title**

**9. Due to COVID-19 have you sent any of the engage materials home for parents to use? and how have they been received? (e.g. online material, printed packs)**

**Question Title**

**10. Please use the text box below to leave any further comments/feedback that you have about project DAIRE. Thank you.**

Bottom of Form

Top of Form

Bottom of Form

| **Project DAIRE: Nourish and Engage Intervention: School Teacher** |
| --- |

**Improving primary school children’s knowledge of and interest in food**

The survey will take approximately 10-15 minutes to complete. The ‘Nourish’ arm of the intervention was based on a food intervention to enhance the current school food environment of participating schools and to increase exposure to Northern Ireland-sourced food. The ‘Nourish’ intervention involved provision of healthy snacks which were provided during the school day and resources were also given to improve the school food environment and school food presentation. The ‘Nourish’ intervention incorporated, tasting Days, cookery equipment and recipes, sensory education and catering for school events. 

The ‘Engage’ arm of intervention was an educational intervention promoting food, agriculture, science and careers associated with the current curriculum in Northern Ireland. The intervention was aimed at P3 and P7 children with lesson plans and classroom activities delivered by teachers, guest speakers and a visit to the farm were also offered.

The ‘Engage’ intervention was based on three topic areas;

Topic 1: Farm to Fork: To increase understanding of where food comes from, how it is grown or produced and how it reaches our plate.

Topic 2: Pleasure on a plate: To enable children to learn about the foods that make up a balanced diet and to increase children’s awareness of buying, preparing and eating food.

Topic 3: Food futures: To enable children to learn about how food production is changing and how new products are being developed.

Activities were developed by the research team and a range of stakeholders to support the lesson plans which included videos, worksheets, games, talks/visits from experts, visits to industry partner workplaces, class trips and practical experiments.

Resources
Videos (Rearing farm, Breeding farm, The hatchery and The Broiler farm, meat video, cheese production, making bread, animal feed), Books (The Right Royal Fishy Tale, sausage story, Johnny love’s milk), games (Say what you see) and worksheets (Eatwell guide, advert templates, who, what why) songs. Sensory education resources, use of recipe books, cookery activities.

**The survey will take approximately 10-15 minutes to complete.**

Top of Form

**Question Title**

**1. Could you please explain your overall experience of project DAIRE? (e.g. what worked well/ not so well?).**

**Question Title**

**2. From your experience do you think project DAIRE is easily integrated into the current Northern Ireland school curriculum? (e.g. educational topic areas, what would really help achieve this? Training?).**

**Question Title**

**3. Since project DAIRE (Sept 18 to June 19) have you explored any topic areas from the intervention into your teaching in this academic year (Sept 2019 to present)? (e.g. Farm to Fork, Pleasure on a plate, Food futures, any changes in delivery, concepts used, has DAIRE had any impact on the way lessons are taught?).**

**Question Title**

**4. Since project DAIRE ended in June 2019 have you used any of the resources provided within your teaching in this academic year (Sept 2019 to present)? (e.g. any changes in delivery, concepts used, has DAIRE had any impact on children’s willingness to try new foods/confidence?).**

If non, please explain your reasons? (e.g. age appropriate, training, further support to implement).

If yes, have you adapted any of the resources?

Which resources do you choose to use and why?

Were the resources easy to incorporate into your teaching plan and were they easy to prepare?

**Question Title**

**5. Are there parts of DAIRE which could not be used last year due to time constraints, which are being used now? (e.g. lesson plans, activities, sensory resources, Tasting days, recipe books).**

**Question Title**

**6. What are your thoughts on healthy snack provision? (e.g. benefits, challenges, best time of the day?).**

**Question Title**

**7. Since project DAIRE ended in June 2019, have you hosted any science or food related events and what did they entail? (e.g. what worked well/ not so well, learning outcomes).If none, what are your reasons?**

**Question Title**

**8. Has your school continued to use any of the following aspects of DAIRE?**

Sensory Education

Enhancement of Dining Area (e.g. table cloths, bunting, menu boards, posters etc)

Enhancement of Food Presentation (e.g. tiered stand, serve ware, fruit and vegetable cutting tools, salad trolleys etc)

Cookery Equipment and Recipes

Healthy Snack Provision (e.g. bread, fruit packs, milk)

Tasting Day

Food Provision for School Event

**Question Title**

**9. Due to COVID-19 have you sent any of the engage intervention materials home for parents to use? and how have they been received? (e.g. online, printed packs).**

**Question Title**

**10. Please use the text box below to leave any further comments/feedback that you have about project DAIRE. (e.g. Information session prior to start date, any future implementation suggestions DAIRE, what did you like/ not like).**

Bottom of Form

Top of Form

Bottom of Form

| **Project DAIRE: Nourish and Engage Intervention: School Catering Representative** |
| --- |

**Improving primary school children’s knowledge of and interest in food**

The ‘Nourish’ arm of the intervention was based on a food intervention to enhance the current school food environment of participating schools and to increase exposure to Northern Ireland-sourced food. The ‘Nourish’ intervention involved provision of healthy snacks which were provided during the school day and resources were also given to improve the school food environment and school food presentation. The ‘Nourish’ intervention incorporated, tasting Days, cookery equipment and recipes, sensory education and catering for school events. 

The ‘Engage’ arm of intervention was an educational intervention promoting food, agriculture, science and careers associated with the current curriculum in Northern Ireland. The intervention was aimed at P3 and P7 children with lesson plans and classroom activities delivered by teachers, guest speakers and a visit to the farm were also offered.

The ‘Engage’ intervention was based on three topic areas;

Topic 1: Farm to Fork: To increase understanding of where food comes from, how it is grown or produced and how it reaches our plate.

Topic 2: Pleasure on a plate: To enable children to learn about the foods that make up a balanced diet and to increase children’s awareness of buying, preparing and eating food.

Topic 3: Food futures: To enable children to learn about how food production is changing and how new products are being developed.

Activities were developed by the research team and a range of stakeholders to support the lesson plans which included videos, worksheets, games, talks/visits from experts, visits to industry partner workplaces, class trips and practical experiments.

Resources
Videos (Rearing farm, Breeding farm, The hatchery and The Broiler farm, meat video, cheese production, making bread, animal feed), Books (The Right Royal Fishy Tale, sausage story, Johnny love’s milk), games (Say what you see) and worksheets (Eatwell guide, advert templates, who, what why) songs. Sensory education resources, use of recipe books, cookery activities.

**The survey will take approximately 10-15 minutes to complete.**

Top of Form

**Question Title**

**1. Is the canteen connected to the school/ part of the school system? (e.g. is there good communication between school canteens and other school departments?**

**Question Title**

**2. What are your thoughts on the primary school canteen environment? (e.g. dining space, queuing, social environment, presentation of food, are there adults present to encourage intake/ monitoring).**

**Question Title**

**3. During project DAIRE what was your involvement? Has this continued since project DAIRE? e.g. Enhancement of canteen (e.g. table cloths, bunting, menu boards, poster etc), food presentation (e.g. tiered stand, serve ware, fruit and vegetable cutting tools, salad trolleys etc), cookery activities, preparation of snack provision (e.g. bread, fruit snack, bread).**

**Question Title**

**4. Do you feel that since the Nourish intervention children have had a willingness to try new foods? Has there been a noticeable change in child food choices?**

**Question Title**

**5. Which DAIRE resources provided have been used to improve the canteen dining area in this academic year (Sept 2019 to present) since project DAIRE finished (June 2019)? (e.g. posters, bunting, platter plates, salad trolleys etc.).**

If none, what are your reasons?

If yes, which ones have been used and what if any improvements have you seen?

**Question Title**

**6. Within the canteen has there been any changes in the presentation of healthy foods and have you used the packs provided to support delivery since project DAIRE? (e.g. If not, what are your reasons and what would really help you implement these?).**

**Question Title**

**7. Since project DAIRE, have you hosted any science or food related events and what did they entail? (e.g. what worked well/ not so well). If none, what are your reasons?**

**Question Title**

**8. Since project DAIRE have any cookery activities been initiated in the canteen setting?**

**Question Title**

**9. What do you feel could be done to support healthy food choices of pupils and improve the school food environment?**

**Question Title**

**10. Please use the text box below to leave an further comments/feedback that you have about project DAIRE. Thank you.**

Bottom of Form

Top of Form

Bottom of Form

| **Project DAIRE: Engage Intervention: School Principal** |
| --- |

**Improving primary school children’s knowledge of and interest in food.**

The ‘Engage’ intervention was an educational intervention promoting food, agriculture, science and agri-food-related careers, developed to align with the current curriculum in Northern Ireland. The intervention was aimed at P3 and P7 children. The intervention contained three topic areas, each with classroom activities and lesson plans to support delivery by teachers. Guest speakers and a trip to the farm were also offered.

The ‘Engage’ intervention was based on three topic areas;

Topic 1: Farm to Fork: To increase understanding of where food comes from, how it is grown or produced and how it reaches our plate.

Topic 2: Pleasure on a plate: To enable children to learn about the foods that make up a balanced diet and to increase children’s awareness of buying, preparing and eating food.

Topic 3: Food futures: To enable children to learn about how food production is changing and how new products are being developed.

Activities were developed by the research team and a range of stakeholders to support the lesson plans which included videos, worksheets, games, talks/visits from experts, visits to industry partner workplaces, class trips and practical experiments.

Resources include: Videos (Rearing farm, Breeding farm, The hatchery and The Broiler farm, meat video, cheese production, making bread, animal feed), Books (The Right Royal Fishy Tale, sausage story, Johnny love’s milk), games (Say what you see) and worksheets (Eatwell guide, advert templates, who, what why) and songs.

**The survey will take approximately 10-15 minutes to complete.**

Top of Form

**Question Title**

**1. Could you please explain your overall experience of project DAIRE?**

**Question Title**

**2. From your experience do you think the engage intervention is easily integrated into the current Northern Ireland school curriculum? (e.g. educational topic areas, lesson plans, what would really help achieve this? further training to implement/time?).**

**Question Title**

**3. Do you have a school food policy in your school? And do you feel that project DAIRE has supported this? (e.g Have you implemented any recent changes to the school food policy? or do you plan to make any changes?**

**Question Title**

**4. Since project DAIRE (Sept 2018 - June 2019) have there been any new science or food related initiatives introduced in your school? (e.g. Breakfast clubs).**

**Question Title**

**5. Are you aware of any components of DAIRE being replicated in this academic year (Sept 2019 - present)?**

**Question Title**

**6. Are there parts of DAIRE which could not be used last year due to time constraints, which are being used now? (e.g. lesson plans or activities).**

**Question Title**

**7. Do you think it would be useful if the research team delivered an introductory project DAIRE information session to all teachers involved in DAIRE prior to the intervention start date? Please explain your reasons.**

**Question Title**

**8. Due to COVID-19 have you sent any of the engage materials home for parents to use? and how have they been received? (e.g. online material, printed packs)**

**Question Title**

**9. What suggestions do you have on future implementation of Engage? (e.g. Topic areas, lesson plans, resources).**

**Question Title**

**10. Please use the text box below to leave any further comments/feedback that you have about project DAIRE. Thank you.**

Bottom of Form

Top of Form

Bottom of Form

| **Project DAIRE: Engage Intervention: School Teacher** |
| --- |

**Improving primary school children’s knowledge of and interest in food.**

The ‘Engage’ intervention was an educational intervention promoting food, agriculture, science and agri-food-related careers, developed to align with the current curriculum in Northern Ireland. The intervention was aimed at P3 and P7 children. The intervention contained three topic areas, each with classroom activities and lesson plans to support delivery by teachers. Guest speakers and a trip to the farm were also offered.

The ‘Engage’ intervention was based on three topic areas;

Topic 1: Farm to Fork: To increase understanding of where food comes from, how it is grown or produced and how it reaches our plate.

Topic 2: Pleasure on a plate: To enable children to learn about the foods that make up a balanced diet and to increase children’s awareness of buying, preparing and eating food.

Topic 3: Food futures: To enable children to learn about how food production is changing and how new products are being developed. 

Activities were developed by the research team and a range of stakeholders to support the lesson plans which included videos, worksheets, games, talks/visits from experts, visits to industry partner workplaces, class trips and practical experiments.

Resources include: Videos (Rearing farm, Breeding farm, The hatchery and The Broiler farm, meat video, cheese production, making bread, animal feed), Books (The Right Royal Fishy Tale, sausage story, Johnny love’s milk), games (Say what you see) and worksheets (Eatwell guide, advert templates, who, what why) and songs.

**The survey will take approximately 10-15 minutes to complete.**

Top of Form

**Question Title**

**1. Could you please explain your overall experience of project DAIRE and your participation in 'Engage'? (e.g. what worked well/not so well?).**

**Question Title**

**2. From your experience do you think the engage intervention is easily integrated into the current Northern Ireland school curriculum? (e.g. educational topic areas, lesson plans, what would really help achieve this? further training to implement/time?).**

**Question Title**

**3. Since project DAIRE (Sept 18-June 19) have you explored any of the topic areas from the engage intervention within your teaching in this academic year (Sept 19-present) ? (e.g. have you made any changes in delivery, have you used any concepts from DAIRE, has DAIRE had any impact on the way lessons are taught?)**

**Question Title**

**4. Since project DAIRE have you used any of the resources provided within your teaching in this academic year (Sept 19-present)?**

If None, please explain your reasons for this?

If Yes, which resources do you choose to use?

Have you adapted any of the resources?

Were the resources easy to incorporate into your teaching?

**Question Title**

**5. Are there parts of DAIRE which could not be used last year due to time constraints, which are being used now?**

**Question Title**

**6. Prior to Covid-19 did you plan to incorporate any of the engage learning materials/resources into your teaching in this academic year (Sept 2019 - present)?**

**Question Title**

**7. Due to COVID-19 have you sent any of the engage intervention materials home for parents to use? and how have they been received? (e.g. online, printed packs).**

**Question Title**

**8. What suggestions do you have on future implementation of DAIRE? (Prompt: lesson plans, taught topics, introduced at the beginning of the school year/ end of the year before to allow preparation time? Funding, further training/support).**

**Question Title**

**9. Do you think it would be useful if the research team delivered an introductory project DAIRE information session to all teachers involved in DAIRE prior to the intervention start date? would you prefer this to be at the end of the previous academic year to allow time for planning? or at the start of the year?**

**Question Title**

**10. Please use the text box below to leave any further comments/ feedback that you have about project DAIRE. Thank you.**

Bottom of Form

Top of Form

Bottom of Form

| **Project DAIRE: Nourish Intervention: School Principal** |
| --- |

**Improving primary school children’s knowledge of and interest in food.**

The ‘Nourish’ intervention was based on enhancing the current school food environment of participating schools and to increase exposure to Northern Ireland-sourced food. The ‘Nourish’ intervention involved provision of healthy snacks which were provided during the school day and resources were also given to improve the school food environment and school food presentation. The ‘Nourish’ intervention incorporated, tasting Days, cookery equipment and recipes, sensory education and catering for school events.

**The survey will take approximately 10-15 minutes to complete.**

Top of Form

**Question Title**

**1. Could you please explain your overall experience of project DAIRE?**

**Question Title**

**2. Do you have a school food policy in your school? And do you feel that project DAIRE has supported this? Have you implemented any changes to your school food policy? And/or have you any plans to make changes to your school food policy?**

**Question Title**

**3. Since project DAIRE ended in June 2019, what elements of the intervention have your school implemented and why? ﻿(healthy snack provision, school canteen resources such as table cloths, posters, menu boards, recipe books, cookery equipment) If none, what are your reasons?**

**Question Title**

**4. Are there parts of DAIRE which could not be used last year due to time constraints which are being used now? (e.g. sensory resources, tasting days, canteen resources such as salad trolleys, posters, menu boards, cookery equipment, recipe books).**

**Question Title**

**5. Since project DAIRE (Sept 2018 - June 2019), have there been any new science or food related initiatives introduced in your school? (e.g. breakfast clubs)**

**Question Title**

**6. What are your thoughts on healthy snack provision? (e.g. impact, benefits, implications, best time of day).**

**Question Title**

**7. If 'N﻿ourish' was to be implemented again in your school what parts of the intervention would you utilise the most and why?**

**Question Title**

**8. Do you think it would be useful if the research team delivered an introductory project DAIRE information session to all teachers and caterers involved in DAIRE prior to the intervention start date?**

**Question Title**

**9. What suggestions do you have on future implementation of Nourish? (e.g. Healthy snack provision, canteen resources, food presentation resources, cookery equipment and recipes, sensory education, tasting day, time and training, funding).**

**Question Title**

**10. Please use the text box below to leave any further comments/feedback that you have about project DAIRE. Thank you.**

Bottom of Form

Top of Form

Bottom of Form

| **Project DAIRE: Nourish Intervention: School Teacher** |
| --- |

**Improving primary school children’s knowledge of and interest in food**

The ‘Nourish’ intervention was based on enhancing the current school food environment of participating schools and to increase exposure to Northern Ireland-sourced food. The ‘Nourish’ intervention involved provision of healthy snacks which were provided during the school day and resources were also given to improve the school food environment and school food presentation. The ‘Nourish’ intervention incorporated, tasting Days, cookery equipment and recipes, sensory education and catering for school events.

**The survey will take approximately 10-15 minutes to complete.**

Top of Form

**Question Title**

**1. Could you please explain your overall experience of project DAIRE?**

**Question Title**

**2. Since project DAIRE (Sept 18 to June 19) have you implemented any parts of the nourish intervention into your teaching in this academic year (Sept 2019 to present)? (e.g. any changes in delivery, concepts used, has DAIRE had any impact on children’s willingness to try new foods/confidence? Sensory education resources, use of recipe books, cookery activities). If no, please explain your reasons?**

**Question Title**

**3. Are there parts of DAIRE which could not be used last year due to time constraints, which are being used now? (e.g. Sensory resources, recipe books, cookery activities, Tasting days).**

**Question Title**

**4. What are your thoughts on healthy snack provision? (e.g. benefits, challenges, best time of the day?).**

**Question Title**

**5. Since project DAIRE ended in June 2019, have you hosted any science or food related events and what did they entail? (e.g. what worked well/ not so well, learning outcomes). If none, what are your reasons?**

**Question Title**

**6. Has your school continued to use any of the following aspects of DAIRE?**

Sensory Education

Enhancement of Dining Area (e.g. table cloths, bunting, menu boards, posters etc)

Enhancement of Food Presentation (e.g. tiered stand, serve ware, fruit and vegetable cutting tools, salad trolleys etc)

Cookery Equipment and Recipes

Healthy Snack Provision (e.g. bread, fruit packs, milk)

Tasting Day

Food Provision for School Events

**Question Title**

**7. What suggestions do you have on future implementation of DAIRE? (e.g. introduced at the beginning of the school year/ end of the year before to allow preparation time? funding).**

**Question Title**

**8. Do you think it would be useful if the research team delivered an introductory Project DAIRE information session to all teachers and caterers involved in DAIRE prior to the intervention start date?**

**Question Title**

**9. What do you miss the most about project DAIRE?**

**Question Title**

**10. Please use the text box below to leave any further comments/feedback that you have about project DAIRE. Thank you.**

Bottom of Form

Top of Form

Bottom of Form

| **Project DAIRE: Nourish Intervention: School Catering Representative** |
| --- |

**Improving primary school children’s knowledge of and interest in food**

The ‘Nourish’ intervention was based on enhancing the current school food environment of participating schools and to increase exposure to Northern Ireland-sourced food. The ‘Nourish’ intervention involved provision of healthy snacks which were provided during the school day and resources were also given to improve the school food environment and school food presentation. The ‘Nourish’ intervention incorporated, tasting Days, cookery equipment and recipes, sensory education and catering for school events.

**The survey will take approximately 10-15 minutes to complete.**

Top of Form

**Question Title**

**1. Is the canteen connected to the school/ part of the school system? (e.g: is there good communication between school canteens and other school departments?**

**Question Title**

**2. What are your thoughts on the primary school canteen environment? (e.g. dining space, queuing, social environment, presentation of food, are there adults present to encourage intake/ monitoring).**

**Question Title**

**3. During project DAIRE what was your involvement? (e.g. Enhancement of canteen (e.g. table cloths, bunting, menu boards, posters), food presentation (e.g. tiered stand, serve ware, fruit and vegetable cutting tools, salad trolleys etc), cookery activities, preparation of snack provision (e.g. bread, fruit packs, milk)? Has this continued since project DAIRE? ).**

**Question Title**

**4. Do you feel that since the Nourish intervention children have had a willingness to try new foods? Has there been a noticeable change in child food choices?**

**Question Title**

**5. Which Nourish resources provided have been used to improve the canteen dining area in this academic year (Sept 2019 to present) since project DAIRE finished (June 2019)? (e.g.posters, bunting, platter plates, salad trolleys etc.).**

If none, please explain your reasons.

If yes, which ones have been used and what if any improvements have you seen?

**Question Title**

**6. Within the canteen has there been any changes in the presentation of healthy foods and have you used the packs provided to support delivery since project DAIRE? If not, what are your reasons and what would really help you implement these?.**

**Question Title**

**7. Since project DAIRE ended in June 2019, have you hosted any science or food related events and what did they entail? (e.g. what worked well/ not so well). If none, why?**

**Question Title**

**8. Since project DAIRE ended in June 2019 have any cookery activities been initiated in the canteen setting?**

**Question Title**

**9. What do you feel could be done to support healthy food choices of pupils and improve the school food environment?**

**Question Title**

**10. Please use the text box below to leave any further comments/feedback that you have about project DAIRE. Thank you.**

Bottom of Form

Top of Form

Bottom of Form

| **Project DAIRE: Control/Delayed Intervention Engage: School Principal** |
| --- |

**Improving primary school children’s knowledge of and interest in food**

The ‘Engage’ intervention was an educational intervention promoting food, agriculture, science and agri-food-related careers, developed to align with the current curriculum in Northern Ireland. The intervention was aimed at P3 and P7 children. The intervention contained three topic areas, each with classroom activities and lesson plans to support delivery by teachers. The delivery materials and resources were given to the school classes at the end of the academic year (2018/2019).

The ‘Engage’ intervention was based on three topic areas;

Topic 1: Farm to Fork: To increase understanding of where food comes from, how it is grown or produced and how it reaches our plate.

Topic 2: Pleasure on a plate: To enable children to learn about the foods that make up a balanced diet and to increase children’s awareness of buying, preparing and eating food.

Topic 3: Food futures: To enable children to learn about how food production is changing and how new products are being developed.

Activities were developed by the research team and a range of stakeholders to support the lesson plans which included videos, worksheets and games.

Resources include: Videos (Rearing farm, Breeding farm, The hatchery and The Broiler farm, meat video, cheese production, making bread, animal feed), Books (The Right Royal Fishy Tale, sausage story, Johnny love’s milk), games (Say what you see) and worksheets (Eatwell guide, advert templates, who, what why) and songs.

**The survey will take approximately 10-15 minutes to complete.**

Top of Form

**Question Title**

**1. Could you please explain your overall experience of project DAIRE?**

**Question Title**

**2. From your experience do you think project DAIRE is easily integrated into the current Northern Ireland school curriculum? (e.g. educational topic areas, lesson plans, what would really help achieve this? further training to implement/time?).**

**Question Title**

**3. Do you have a school food policy in your school? And do you feel that project DAIRE has supported this? (Prompt: Have you implemented any recent changes to the school food policy? or do you plan to make any changes?**

**Question Title**

**4. Since project DAIRE (Sept 2018 - June 2019) have there been any new science or food related initiatives introduced in your school? (e.g. Breakfast clubs).**

**Question Title**

**5. Are you aware of any components of DAIRE being replicated in your school in this academic year (Sept 2019 – Present)?**

**Question Title**

**6. Are there parts of DAIRE which could not be used last year due to time constraints, which are being used now? (e.g. lesson plans or activities).**

**Question Title**

**7. Do you think it would be useful if the research team delivered an introductory project DAIRE information session to all teachers involved in DAIRE prior to the intervention start date? Please explain your reasons.**

**Question Title**

**8. Due to COVID-19 have you sent any of the engage materials home for parents to use? and how have they been received? (e.g. online material, printed packs)**

**Question Title**

**9. What suggestions do you have on future implementation of Engage? (e.g. Topic areas, lesson plans, resources).**

**Question Title**

**10. Please use the text box below to leave any further comments/feedback that you have about project DAIRE. Thank you.**

Bottom of Form

Top of Form

Bottom of Form

| **Project DAIRE: Control/ Delayed Intervention Engage: School Teacher** |
| --- |

**Improving primary school children’s knowledge of and interest in food**

The ‘Engage’ intervention was an educational intervention promoting food, agriculture, science and agri-food-related careers, developed to align with the current curriculum in Northern Ireland. The intervention was aimed at P3 and P7 children. The intervention contained three topic areas, each with classroom activities and lesson plans to support delivery by teachers. The delivery materials and resources were given to the school classes at the end of the academic year (2018/2019).

The ‘Engage’ intervention was based on three topic areas;

Topic 1: Farm to Fork: To increase understanding of where food comes from, how it is grown or produced and how it reaches our plate.

Topic 2: Pleasure on a plate: To enable children to learn about the foods that make up a balanced diet and to increase children’s awareness of buying, preparing and eating food.

Topic 3: Food futures: To enable children to learn about how food production is changing and how new products are being developed.
 
Activities were developed by the research team and a range of stakeholders to support the lesson plans which included videos, worksheets and games.

Resources
Videos (Rearing farm, Breeding farm, The hatchery and The Broiler farm, meat video, cheese production, making bread, animal feed), Books (The Right Royal Fishy Tale, sausage story, Johnny love’s milk), games (Say what you see) and worksheets (Eatwell guide, advert templates, who, what why) and songs.

**The survey will take approximately 10-15 minutes to complete.**

Top of Form

**Question Title**

**1. Could you please explain your overall experience of Project DAIRE?**

**Question Title**

**2. From your experience do you think the engage intervention is easily integrated into the current Northern Ireland school curriculum? (e.g. educational topic areas, lesson plans, what would really help achieve this? further training to implement/time?).**

**Question Title**

**3. Since project DAIRE (end of the academic year 2018/19) have you explored any of the topic areas from the engage intervention in your teaching in this academic year (Sept 2019 to present)? (e.g. Farm to Fork, Pleasure on a plate, Food futures).**

If none, please explain your reasons?

If yes, please explain which ones you have utilised

**Question Title**

**4. Since project DAIRE (end of academic year 2018/19) have you used any of the resources provided within your teaching in this academic year (Sept 2019 to present)?**

If none, please explain your reasons? (e.g. age appropriate, training, further support to implement, time)

If yes, Have you adapted any of the materials?

Which resources do you choose to use and why?

Were the resources easy to incorporate into your teaching plan?

**Question Title**

**5. Are there parts of DAIRE which could not be used last year due to time constraints, which are being used in this academic year (Sept 2019 - present?**

**Question Title**

**6. Prior to COVID-19 did you plan to incorporate any of the engage learning materials/ resources into your teaching in this academic year (Sept 2019 - present)?**

**Question Title**

**7. Due to COVID-19 have you sent any of the engage intervention materials home for parents to use? And how have they been received? (Online material, Printed packs).**

**Question Title**

**8. What suggestions do you have on future implementation of DAIRE? (Prompt: lesson plans, topics, introduced at the beginning of the school year/ end of the year before to allow preparation time? Funding, further training/support).**

**Question Title**

**9. Do you think it would be useful if the research team delivered an introductory Project DAIRE information session to all teachers involved in DAIRE prior to the delivery of resources?**

**Question Title**

**10. Please use the text box below to leave any further comments/ feedback that you have about project DAIRE. Thank you.**

Bottom of Form

Top of Form

Bottom of Form
