# Supplementary material for: Process evaluation of Project Daire: a food environment intervention that impacted food knowledge, wellbeing and dietary habits of primary school children
Source: BMC Public Health. 2025 Feb 6;25:486. doi: 10.1186/s12889-025-21628-4 (PMC11800617; doi:10.1186/s12889-025-21628-4)
Supplement: Supplementary file 7 [file 12889_2025_21628_MOESM7_ESM.docx]

**Additional File 7: Flow Diagram showing DAIRE intervention recruitment**

**
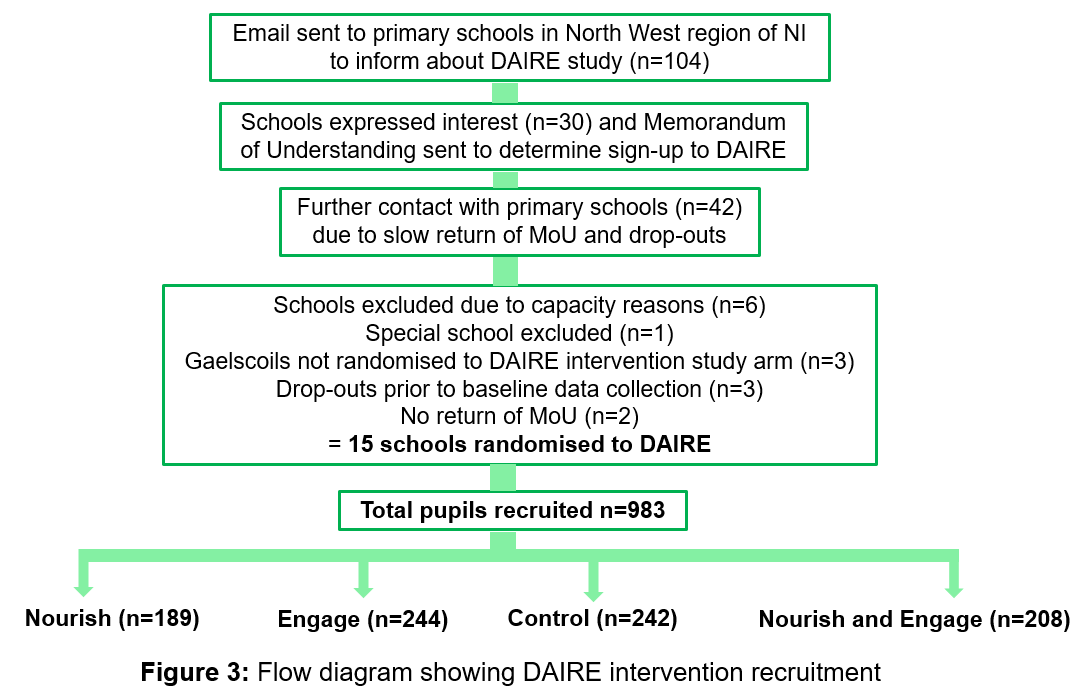
**

In terms of DAIRE recruitment, an initial contact email was sent to primary schools within the Derry City and Strabane District Council area (n=104) in January 2019, as well as schools who were contacts of/in contact with DAIRE researchers previously.

Following email contact, a MoU was sent to interested primary schools (n=30) with return demonstrating formal sign up on a ‘first-come first-serve’ basis.

A small number of schools dropped out (n=3) prior to baseline data collection due to reasons unrelated to DAIRE i.e., financial/school pressures. At the same time, slow MoU return (n=2 did not return) resulted in contacting a further 42 schools about the DAIRE intervention in late January/early February 2019, leading to 146 schools contacted in total.

Following this, schools (n=6) were excluded due to the DAIRE research team capacity issues, a special school (n=1) was provided with intervention materials but excluded due to not meeting the study eligibility criteria and Gaelscoils (n=3) took part in the study but were not randomised, due to time and resource-constraints regarding translation of materials from English to Irish language. Consequently, a total of 15 schools were recruited and randomised within DAIRE. Finally, of the 146 schools contacted, 30 expressed interest and were sent a MoU, indicating a 20.5% interest response rate from schools.

Power calculations were conducted for this factorial design intervention to compare ‘Nourish; versus not Nourish’ and ‘Engage; versus not Engage’ and indicated that the trial required 960 pupils, therefore recruiting 983 pupils ensured DAIRE was an appropriately powered trial.

**Follow-Up Questionnaires**

The DAIRE team planned to conduct follow-up interviews with school staff about their views and experiences of DAIRE but were unable to do so due to COVID-19-related school closures. Instead, one year after completion of the trial, follow-up questionnaires designed for school representatives (principals, teachers and/or caterers) were sent to all participating primary schools approximately 1 year after the end of the interventions and responses were received by 5 schools, giving a response rate of 33% (5 principals, 6 teachers and 3 caterers). This questionnaire collected quantitative and qualitative data on the impact of DAIRE on individual school food policies, healthy snack provision, use of DAIRE components after the intervention period, and suggestions for future implementation of DAIRE.
