# Supplementary material for: Process evaluation of Project Daire: a food environment intervention that impacted food knowledge, wellbeing and dietary habits of primary school children
Source: BMC Public Health. 2025 Feb 6;25:486. doi: 10.1186/s12889-025-21628-4 (PMC11800617; doi:10.1186/s12889-025-21628-4)
Supplement: Supplementary file 8 [file 12889_2025_21628_MOESM8_ESM.docx]

**Additional File 8 – Engage topics and lessons delivered**

**Table 1:** The lessons delivered within each of the three Engage topics at school level

| **Engage schools (n=7)** | **Topic 1: Farm to Fork** | | | | **Number of lessons delivered *n* (%)** | **Topic 2: Pleasure on a Plate** | | | | **Number of lessons delivered *n* (%)** | **Topic 3: Food Futures** | | | | | | | | **Number of lessons delivered *n* (%)** |
| --- | --- | --- | --- | --- | --- | --- | --- | --- | --- | --- | --- | --- | --- | --- | --- | --- | --- | --- | --- |
|  | *Animal Welfare* | *Food Scribblers* | *Food Stories* | *Johnny Loves Milk* |  | *Growing* | *Portion Size* | *Seasonality* | *Sensory Scientists* |  | *Favourite Food* | *Food Ideas* | *In the restaurant* | *Building ideas* | *Marketing* | *Advertising* | *Great Teams* | *Business Planning* |  |
| **C** | ✓ | ✓ | ✓ | X | 3(75) | X | ✓ | ✓ | X | 2(50) | ✓ | ✓ | ✓ | X | X | X | X | X | 3(37.5) |
| **D** | X | ✓ | ✓ | ✓ | 3(75) | ✓ | ✓ | X | X | 2(50) | ✓ | ✓ | X | X | ✓ | ✓ | X | X | 4(50) |
| **O** | ✓ | ✓ | X | ✓ | 3(75) | ✓ | ✓ | X | X | 2(50) | ✓ | ✓ | ✓ | X | X | ✓ | X | X | 4(50) |
| **G** | X | ✓ | ✓ | ✓ | 3(75) | ✓ | ✓ | ✓ | ✓ | 4(100) | X | X | X | X | X | X | X | X | 0(0) |
| **L** | ✓ | ✓ | ✓ | ✓ | 4(100) | X | X | X | X | 0(0) | ✓ | ✓ | ✓ | ✓ | ✓ | X | X | X | 5(62.5) |
| **P** | ✓ | ✓ | ✓ | ✓ | 4(100) | X | X | X | X | 0(0) | ✓ | ✓ | ✓ | ✓ | ✓ | X | X | X | 5(62.5) |
| **R** | ✓ | ✓ | ✓ | ✓ | 4(100) | ✓ | ✓ | ✓ | X | 3(75) | ✓ | ✓ | X | X | X | X | X | X | 2(25) |
| **Mean Dose delivered per topic (%)** | Topic 1: 85.7 | | | | | Topic 2: 46.4 | | | | | Topic 3: 41.1 | | | | | | | | |
| **Mean (SD) Number of lessons delivered per topic:** Topic 1: 3.4(0.53) Topic 2:1.9(1.46) Topic 3: 3.3(1.8) | | | | | | | | | | | | | | | | | | | |

*During DAIRE development, following early teacher feedback on the large volume of Engage content, the research team agreed with Engage partner schools that a core minimum Engage delivery should consist of of n=5 lessons with associated activities and n=1 talk from a guest speaker should be completed for the intervention.*
