# Supplementary material for: Process evaluation of Project Daire: a food environment intervention that impacted food knowledge, wellbeing and dietary habits of primary school children
Source: BMC Public Health. 2025 Feb 6;25:486. doi: 10.1186/s12889-025-21628-4 (PMC11800617; doi:10.1186/s12889-025-21628-4)
Supplement: Supplementary file 9 [file 12889_2025_21628_MOESM9_ESM.docx]

**Additional File 9 – Engage guest speakers, visits and activities utilised**

**Table 1:** Engage guest speakers, visits and activities utilised at school level

| **Engage Schools (n=7)** | **Guest Speakers** | | | | **Number of guest speakers utilised per school *n* (%)** | **Visits** | | | | **Number of food industry visits completed per school *n* (%)** | **Activities** | | **Number of activities completed per school *n* (%)** |
| --- | --- | --- | --- | --- | --- | --- | --- | --- | --- | --- | --- | --- | --- |
|  | *Beef and where does food come from?* | *Dairy and milking technology* | *Portion Size* | *Packaging* |  | *FIP1* | *FIP2I* | *FIP3* | *FIP4* |  | *Mobile Petting farm* | *Dragons Den* |  |
| **C** |  |  |  |  | 4(100) | X | X | X | X | 0(0) |  | X | 1(50) |
| **D** |  | X |  | X | 2(50) | X | X | X | X | 0(0) |  | X | 1(50) |
| **O** |  |  |  |  | 4(100) | X | X | X | X | 0(0) |  | X | 1(50) |
| **G** |  |  |  |  | 4(100) | X | X | X | X | 0(0) | X | X | 0(0) |
| **L** |  |  |  |  | 4(100) |  | X | X | X | 1(25) |  | X | 1(50) |
| **P** |  |  | X | X | 2(50) |  |  | X |  | 3(75) |  | X | 1(50) |
| **R** | X |  | X | X | 1(25) | X | X | X | X | 0(0) |  | X | 1(50) |
| **Mean Dose delivered (%)** | Guest speakers: 75 | | | |  | Visits: 14.3 | | | |  | Activities: 42.9 | |  |
| **Mean (SD)** Number of guest speakers: 3(1.29) Number of visits completed: 0.6(1.13) Number of activities completed:0.9(0.38) | | | | | | | | | | | | | |
