# Supplementary material for: Process evaluation of Project Daire: a food environment intervention that impacted food knowledge, wellbeing and dietary habits of primary school children
Source: BMC Public Health. 2025 Feb 6;25:486. doi: 10.1186/s12889-025-21628-4 (PMC11800617; doi:10.1186/s12889-025-21628-4)
Supplement: Supplementary file 10 [file 12889_2025_21628_MOESM10_ESM.docx]

**Additional File 10 – ETEQ data on Engage resources utilised Table 1:** ETEQ data on Engage resources utilised at both school and key stage level

|  | | **Schools who used resource *n* (%)** | **Mean (SD) resources used within topics at school level** | **Mean number of KS1 classes who used resource *n* (%)**  **(n=9)** | **Mean number of KS2 classes who used resource *n* (%) (n=2)** | **Mean % of each topic per Key Stage**  **KS1 KS2** | |
| --- | --- | --- | --- | --- | --- | --- | --- |
| **Topic 1: Farm to Fork** | | | | | | | |
| *Animal welfare* | ‘A right royal fishy tail’ book | 5(71.4) | 11.8(1.95) | 7(77.8) | 1(50) | 79.4 | 28.6 |
|  | Video links with lesson plans | 6(85.7) |  | 8(88.9) | 1(50) |  |  |
|  | KS1/KS2 slide deck | 6(85.7) |  | 8(88.9) | 2(100) |  |  |
| *Food scribblers* | Cheese production video | 7(100) |  | 8(88.9) | 0(0) |  |  |
|  | Meat video | 7(100) |  | 8(88.9) | 0(0) |  |  |
|  | Sausage story | 7(100) |  | 8(88.9) | 0(0) |  |  |
|  | FIP4 game | 7(100) |  | 7(77.8) | 0(0) |  |  |
|  | Video links with lesson plan | 7(100) |  | 8(88.9) | 0(0) |  |  |
| *Food stories* | ‘Mummy’s brown bread’ video | 5(71.4) |  | 6(66.7) | 1(50) |  |  |
|  | FIP2 video | 5(71.4) |  | 5(55.6) | 0(0) |  |  |
|  | ‘Fakeaway’ video | 5(71.4) |  | 6(66.7) | 0(0) |  |  |
| *Johnny Loves Milk* | ‘Johnny loves milk’ book | 6(85.7) |  | 8(88.9) | 1(50) |  |  |
|  | Pictures for food chain task | 6(85.7) |  | 7(77.8) | 1(50) |  |  |
|  | FIP5 animal feed production video | 5(71.4) |  | 6(66.7) | 1(50) |  |  |
| **Topic 2: Pleasure on a Plate** | | | |  |  |  | |
| *Growing* | Video links with lesson plan: 5-a-day | 2(28.5) | 5.4(2.99) | 4(44.4) | 1(50) | 34.1 | 33.3 |
|  | Seeds to grow cress | 2(28.5) |  | 3(33.3) | 0(0) |  |  |
|  | Experiment sheet | 3(42.8) |  | 4(44.4) | 0(0) |  |  |
|  | Mushrooms slide | 2(28.5) |  | 3(33.3) | 0(0) |  |  |
|  | Video links with lesson plans | 3(42.8) |  | 4(44.4) | 1(50) |  |  |
| *Portion size* | Eat well guide | 6(85.7) |  | 7(77.8) | 2(100) |  |  |
|  | Eat well guide slides | 5(71.4) |  | 6(66.7) | 2(100) |  |  |
|  | Portion size slides | 4(57.1) |  | 5(55.6) | 2(100) |  |  |
| *Seasonality* | Food miles video link | 1(14.2) |  | 1(11.1) | 0(0) |  |  |
|  | Food maps | 3(42.8) |  | 2(22.2) | 1(50) |  |  |
|  | Seasonal veg calendar | 3(42.8) |  | 2(22.2) | 1(50) |  |  |
|  | Seasonality clock | 2(28.5) |  | 2(22.2) | 0(0) |  |  |
| *Sensory scientists* | Taste Task-lab coats | 1(14.2) |  | 1(11.1) | 0(0) |  |  |
|  | Homework Taste Task/word bank | 0(0) |  | 0(0) | 0(0) |  |  |
|  | Taste slides | 2(28.5) |  | 2(22.2) | 0(0) |  |  |
| **Topic 3: Food Futures** | | | |  |  |  | |
| *Favourite food* | Nutritionist video | 4(57.1) | 2.5(2.64) | 4(44.4) | 1(50) | 28.9 | 15 |
|  | Eat Well Guide | 5(71.4) |  | 6(66.7) | 1(50) |  |  |
| *Food ideas* | Chef video | 4(57.1) |  | 4(44.4) | 0(0) |  |  |
|  | Who, what, why template | 4(57.1) |  | 4(44.4) | 0(0) |  |  |
| *In the restaurant* | Restaurateur video | 3(42.8) |  | 3(33.3) | 1(50) |  |  |
| *Building ideas* | N/A | N/A |  | N/A | N/A |  |  |
| *Marketing* | Marketer video | 1(14.2) |  | 1(11.1) | 0(0) |  |  |
|  | Marketing template | 2(28.5) |  | 2(22.2) | 0(0) |  |  |
| *Advertising* | N/A | N/A |  | N/A | N/A |  |  |
| *Great teams* | Video | 1(14.2) |  | 1(11.1) | 0(0) |  |  |
|  | Marshmallows/lollypop sticks | 0(0) |  | 0(0) | 0(0) |  |  |
| *Business planning* | Food futures videos | 1(14.2) |  | 1(11.1) | 0(0) |  |  |
