# Supplementary material for: Process evaluation of Project Daire: a food environment intervention that impacted food knowledge, wellbeing and dietary habits of primary school children
Source: BMC Public Health. 2025 Feb 6;25:486. doi: 10.1186/s12889-025-21628-4 (PMC11800617; doi:10.1186/s12889-025-21628-4)
Supplement: Supplementary file 11 [file 12889_2025_21628_MOESM11_ESM.docx]

**Additional File 11 – Figure showing the risks of contamination in DAIRE (1)**


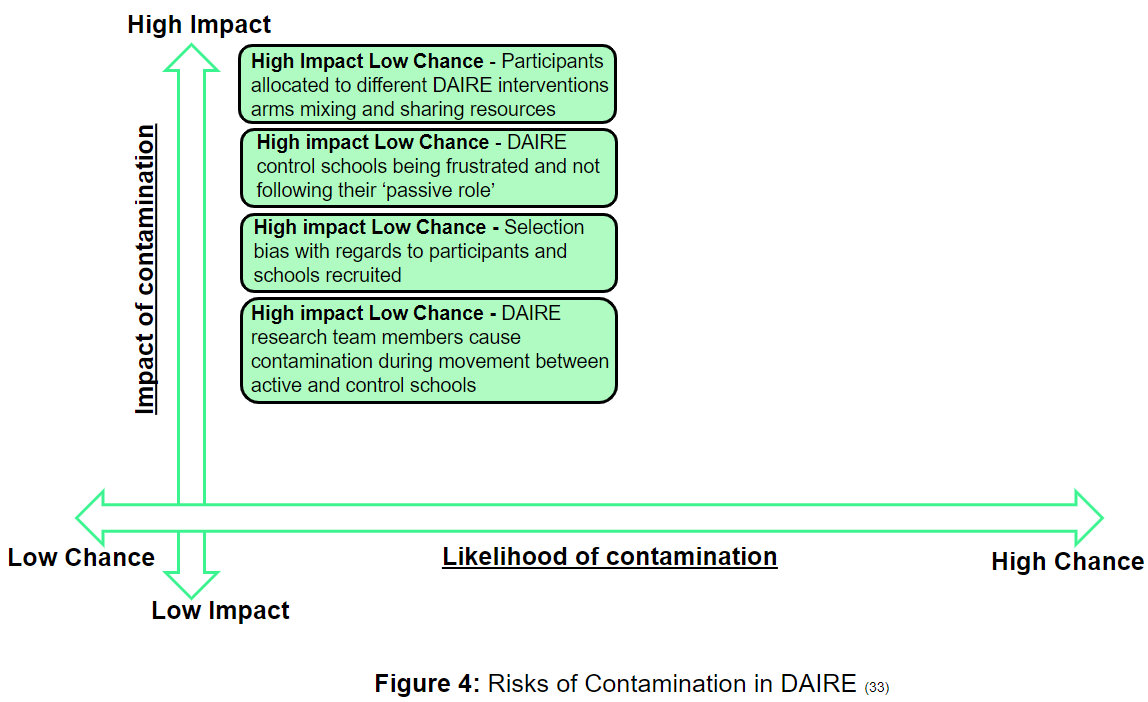


Reference:

(1) Stamp E, Schofield H, Roberts VL, Burton W, Collinson M, Stevens J, et al. Contamination within trials of community-based public health interventions: lessons from the HENRY feasibility study. Pilot and Feasibility Studies. 2021;7(1):88.
